# Systematic review and meta-analysis of humoral immunity proteins and mortality in sepsis

**DOI:** 10.1186/s13054-025-05758-0

**Published:** 2025-12-22

**Authors:** Antoine Villa, Fiona Dewar, Walter Pisciotta, Ankit Rai, Sven Kerneis, Gül Batum, Tom McDonnell, Marie Scully, Timothy D. McHugh, Kai Hilpert, Derek Gilroy, Aline de Nooijer, Mihai G. Netea, Morten Hedetoft, Jesús F. Bermejo-Martin, Masayuki Akatsuka, Corina C. Heinz, Fabienne Venet, Guillaume Monneret, Jennifer Meessen, Tzu Hsuan Cheng, Ming Zhang, Pietro Caironi, Evangelos J. Giamarellos-Bourboulis, Mari C. de la Torre Terrón, Henning Ebelt, Emma Rademaker, Mikael Bodelsson, Jonas Tverring, Yuxin Mi, Julian C. Knight, Merry L. Lindsey, Raymond J. Langley, Stephen F. Kingsmore, Dave Brealey, Mervyn Singer, Nishkantha Arulkumaran

**Affiliations:** 1 Bloomsbury Institute of Intensive Care Medicine, University College London, London, UK; 2 Department of Anesthesia and Intensive Care, IRCCS-ISMETT, Palermo, Italy; 3 Institute of Inflammation and Repair, University College London, London, UK; 4 Department of Haematology, UCLH and Haematology Programme-NIHR UCLH/UCL BRC, London, UK; 5 Centre for Clinical Microbiology, Division of Infection and Immunity, University College London, London, UK; 6 Institute for Infection and Immunity, St George’s, University of London, London, UK; 7 Centre for Clinical Pharmacology, University College London, London, UK; 8 Department of Internal Medicine, Radboud University Medical Center, Nijmegen, Netherlands; 9 Department of Anaesthesia, Rigshospitalet, University of Copenhagen, Copenhagen, Denmark; 10 Universidad de Salamanca & Instituto de Investigación Biomédica de Salamanca (IBSAL) & CIBERES, Instituto de Salud Carlos III, Madrid, Spain; 11 Department of Biochemistry, Department of Intensive Care Medicine, Sapporo Medical University School of Medicine, Sapporo, Japan; 12 Department of Clinical Research and Development, Biotest, Dreieich, AG Germany; 13 CIRI, Centre International de Recherche en Infectiologie, Inserm U1111, CNRS UMR5308, École Normale Supérieure de Lyon, Université Claude Bernard Lyon 1, Université de Lyon, Lyon, F-69007 France; 14 Laboratoire d’Immunologie, Hôpital E. Herriot, Hospices Civils de Lyon, Lyon, France; 15 Department of Acute, Brain and Cardiovascular Injury, Institute for Pharmacological Research Mario Negri IRCCS, Milan, Italy; 16 Department of Oncology, University of Turin, Turin, Italy; 17 Department of Internal Medicine, Medical School, National and Kapodistrian University of Athens, Athens, Greece; 18 Department of Intensive Care Medicine, Hospital Universitari de Mataró, Consorci Sanitari del Maresme, Mataró, Spain; 19 Department of Internal Medicine III, University Hospital Halle (Saale), Catholic Hospital St. Johann Nepomuk, Halle, Erfurt, Germany; 20 Department of Intensive Care Medicine, University Medical Center Utrecht, Utrecht, Netherlands; 21 Department of Clinical Sciences, Lund University, Lund, Sweden; 22 Centre for Human Genetics, University of Oxford, Oxford, UK; 23 Department of Applied Education, Department of Biomedical Sciences, Meharry Medical College, Nashville, TN USA; 24 Research Service, Nashville VA Medical Center, Nashville, TN USA; 25 Department of Pharmacology, Whiddon College of Medicine, University of South Alabama, Mobile, AL USA; 26 Rady Children’s Institute for Genomic Medicine, Rady Children’s Hospital, San Diego, CA USA; 27 Department of Anesthesiology, SUNY Downstate Health Sciences University, Brooklyn, NY USA; 28 Department of Cell Biology, SUNY Downstate Health Sciences University, Brooklyn, NY USA

**Keywords:** Sepsis, Humoral immunity, Complement, Immunoglobulins, Mortality, ICU

## Abstract

**Purpose:**

Humoral immunity proteins—immunoglobulins, complement proteins, and antimicrobial peptides—have key antimicrobial and immunomodulatory functions in sepsis. We hypothesised that their circulating levels are lower in non-survivors, potentially resulting in impaired bacterial clearance and persistent or recurrent infections.

**Methods:**

We performed a systematic review and meta-analysis evaluating differences in humoral immunity proteins between survivors and non-survivors in adult patients with sepsis. PubMed and Embase were searched without date restrictions. Random-effects meta-analyses were used to estimate pooled standardised mean differences (SMD) with 95% confidence intervals (CI). Sensitivity analyses included data from the MIMIC-IV ICU database, and further supplemented by three proteomic studies.

**Results:**

Thirty-six studies including 6,330 patients were analysed. Thirteen reported on immunoglobulins, 17 on complement proteins, and 7 on the antimicrobial peptide heparin-binding protein (HBP). Survivors had significantly higher levels of complement proteins C3 (SMD 0.53 [0.07–0.99]) and C4 (SMD 0.51 [0.09–0.94]) compared to non-survivors. Conversely, C4a (SMD − 1.17 [–1.77 to − 0.56]) and IgA (SMD − 0.21 [–0.39 to − 0.03]) were significantly lower in survivors. No differences were found for IgG (SMD 0.00 [–0.18 to 0.18]), IgM (SMD − 0.02 [–0.13 to 0.08]), C5, C5a, or HBP. Sensitivity analyses using MIMIC-IV (*n* = 2,452) and proteomic datasets supported these findings. Proteomic data revealed early depletion of classical complement components (C3, C4B) and regulatory proteins in non-survivors.

**Conclusion:**

Sepsis non-survivors exhibit lower C3 and C4 levels and higher C4a, consistent with complement activation and/or depletion. Complement proteins may serve as potential biomarkers and therapeutic targets in sepsis.

**Supplementary Information:**

The online version contains supplementary material available at 10.1186/s13054-025-05758-0.

## Background

Sepsis, defined as life-threatening organ dysfunction caused by a dysregulated host response to infection, accounts for nearly 20% of all global deaths [1, 2]. The immune response in sepsis evolves from an early hyperinflammatory phase to a compensatory immunosuppressive phase [3]. While considerable attention has been paid to the cellular aspects of this immune dysfunction [4, 5], the contribution of humoral immunity mediated by soluble plasma proteins remains incompletely understood.

Humoral immunity proteins comprise immunoglobulins, complement proteins, and antimicrobial peptides (AMPs), which exert both antimicrobial and immunomodulatory effects. Immunoglobulin-mediated activation of the classical complement pathway leads to sequential cleavage of complement proteins, culminating in the formation of the membrane attack complex (MAC) that disrupts bacterial membranes [6, 7]. Complement and immunoglobulins also promote opsonisation and phagocytosis [8]. AMPs—including defensins, cathelicidins, and pentraxins—interact with microbial membranes and modulate host immune responses with broad-spectrum activity and low toxicity [9].

We hypothesised that lower circulating levels of humoral immunity proteins would be observed in non-survivors of sepsis, reflecting excessive activation, impaired synthesis, and/or redistribution. To address this, we conducted a systematic review and meta-analysis of published studies comparing humoral immunity protein levels between sepsis survivors and non-survivors. Additionally, we explored levels of immunoglobulins and complement proteins in the MIMIC-IV ICU database [10], and further validated findings through secondary analysis of three independent proteomic datasets.

## Methods

### Study design

We conducted a systematic review and meta-analysis in accordance with the PRISMA 2020 guidelines, registered on PROSPERO (CRD42025542278). The primary objective was to assess the association between circulating levels of humoral immunity proteins and mortality in adult patients with sepsis. In addition to pooled analysis of published studies, we performed two complementary exploratory analyses: (1) a retrospective cohort study using the MIMIC-IV database, and (2) secondary analysis of proteomic datasets from three published studies.

### Search strategy

A systematic literature search of PubMed and Embase was conducted in January 2025, without date or language restrictions. The following Boolean search terms were used:

(IVIg OR immunoglobulin OR complement OR C3 OR C4 OR antimicrobial peptide OR CRP OR C reactive protein OR Pentraxin OR cathelicidin OR defensin OR LL-37) AND (sepsis OR critical illness OR critically ill OR Intensive Care OR critical care) AND (survival OR mortality OR secondary infection) NOT (animal OR COVID OR SARS-CoV).

### Eligibility criteria

We included observational or interventional studies that reported levels of humoral immunity proteins—complement components, immunoglobulins, or antimicrobial peptides—in adult patients (≥ 18 years old) with sepsis, stratified by survival status. Only studies reporting measurements within the first 24 h of ICU or hospital admission were eligible. Studies focused solely on COVID-19 or conducted in animal models were excluded, although mixed sepsis cohorts that may have included a small number of patients with viral pneumonia were retained. Proteomic studies were eligible for exploratory inclusion if they quantified relevant proteins and provided stratification by survival. Authors were contacted to obtain raw or processed data when necessary.

### Study selection and data extraction

Titles and abstracts were screened independently by four investigators (GB, SK, WP, AR), with discrepancies resolved by a fifth reviewer (AV). Data extraction was performed independently by two reviewers (AV, NA) using a pre-specified form. Extracted data included study characteristics, protein levels in survivors and non-survivors, assay methods, clinical context, and mortality endpoints. When data were reported in non-metric units (e.g., mmol/L), conversions to mg/dL were performed using molecular weights from standard biochemical references.

### Assessment of study quality

The Newcastle–Ottawa Scale (NOS) was used to assess the quality of studies. The NOS evaluates three domains: selection of the cohort (maximum 4 points), comparability of the groups (maximum 2 points), and quality of outcome assessment (maximum 3 points), for a total score ranging from 0 to 9 [11]. The reference population was adult ICU patients, outcome 28-day mortality. Studies scoring ≥ 7 were considered high quality, scores of 4–6 moderate quality, and ≤ 3 low quality.

### Outcomes

The primary outcome was the difference in circulating levels of humoral immunity proteins between survivors and non-survivors of sepsis. In cases where 28-day mortality was not reported, 30-day, ICU or hospital mortality was used.

### Sensitivity and secondary analyses

To test the robustness and extend the scope of our findings, we performed additional sensitivity and exploratory analyses.

First, we repeated the meta-analyses restricted to published studies that explicitly reported protein measurements within the first 24 h of ICU or hospital admission.

Second, we performed a pooled sensitivity analysis incorporating data from the MIMIC-IV (Medical Information Mart for Intensive Care IV) database [10] (access certification ID: 63989768). MIMIC-IV contains de-identified ICU admissions at Beth Israel Deaconess Medical Center between 2008 and 2019. Adult patients with sepsis, defined by Sepsis-3 criteria, and with available 28-day mortality data were included. For this pooled analysis, only patients with measurements of C3, C4, IgA, IgG, or IgM obtained within the first 24 h of ICU admission were eligible. Because of indication bias for protein testing, these data were considered hypothesis-generating and not included in the main meta-analysis.

Third, we conducted secondary analyses. In MIMIC-IV, we further explored temporal trajectories of immunoglobulins and complement proteins measured from admission up to day 5. In addition, we analysed three independent proteomic or glycoproteomic datasets [12–14] reporting differential expression of humoral immunity proteins in sepsis survivors and non-survivors. Raw data were log2-transformed and visualised using volcano plots and heatmaps; only proteins with *p* < 0.05 were considered significant. Two-way ANOVA was used to assess temporal changes and interactions by survival status.

### Statistical analysis

Meta-analyses were performed using random-effects models with inverse variance weighting to calculate standardised mean differences (SMDs) and 95% confidence intervals (CIs) between survivors and non-survivors. Where necessary, means and standard deviations were derived from medians and interquartile ranges using Cochrane-recommended formulas. Between-study heterogeneity was assessed using the *I²* statistic (25%, 50%, and 75% representing low, moderate, and high heterogeneity, respectively).

Sensitivity analyses included restriction to studies measuring proteins within 24 h of ICU admission and pooled analyses incorporating data from the MIMIC-IV cohort. In the MIMIC-IV dataset, comparisons between survivors and non-survivors were made using Mann–Whitney U tests for continuous variables and χ² or Fisher’s exact tests for categorical variables, as appropriate. Temporal changes in humoral protein levels (days 0–1 vs. 2–5) were evaluated using paired Wilcoxon signed-rank tests within groups and two-way ANOVA with interaction terms (time × survival) to assess longitudinal differences.

In the proteomic datasets, log₂-transformed protein intensities were compared using unpaired two-sample t-tests, and volcano plots were generated to visualize differential abundance (log₂ fold change vs. –log₁₀ *p*-value). Two-way ANOVA was also applied to identify significant effects of time, survival status, and their interaction.

All *p*-values were two-sided, and *p* < 0.05 was considered statistically significant. Analyses were conducted using R (v4.1.2; R Core Team, 2021)—including the *meta*, *metafor*, and *dplyr* packages—and GraphPad Prism (v9.0, GraphPad Software, CA).

## Results

### Meta-analysis

**Fig. 1:**
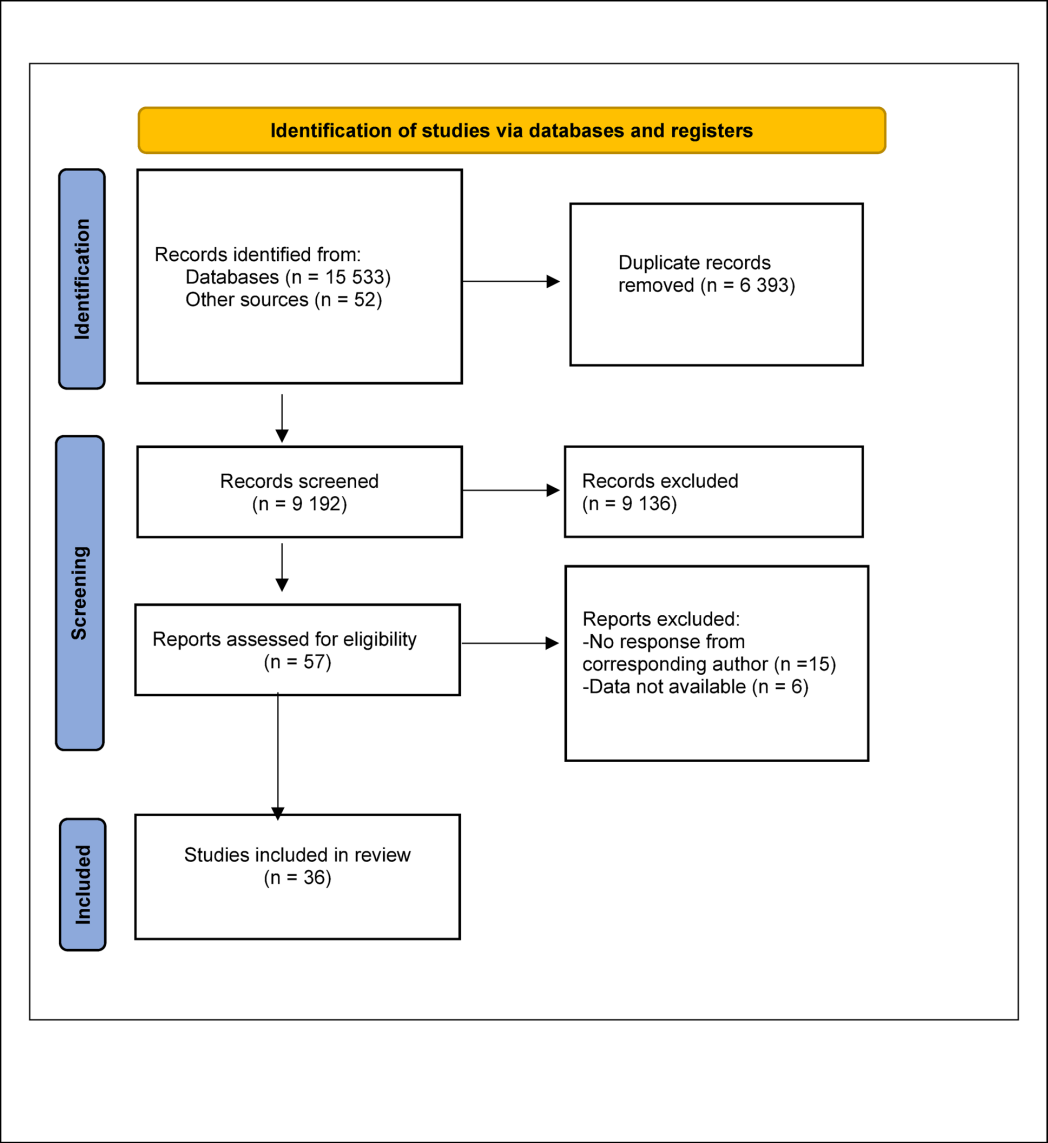
PRISMA flowchart. Flowchart of included and excluded trials

A total of 15,533 articles were identified through database searches, and a further 52 articles by hand search. After removal of 6,393 duplicates, 9,192 records were screened based on titles and abstracts. Of these, 9,136 were excluded. Fifty-seven full-text articles were assessed for eligibility, including 13 identified through hand search [15–27]. Twenty-one articles were excluded; 15 [23, 28–40] due to non-response from corresponding authors following request for data, and 6 [41–46] where data were not available. Thirty-six studies were included in the final review (Fig. 1) [15–27, 47–69]. Data were obtained from the corresponding authors in 15 [18, 21–24, 27, 47, 60–62, 64–67, 69] of these studies.

Three studies [12–14] reporting relative differences in levels of humoral immunity proteins between sepsis survivors and non-survivors using proteomic and/or mass spectrometry techniques were included. As levels of proteins were reported as relative differences rather than absolute values, it was not possible to include these data in the quantitative meta-analysis.

#### 1a. Complement proteins

Seventeen studies [24–27, 47–59], including 2,222 patients with an overall 36% mortality, reported circulating complement protein levels. The most frequently measured components were C3 (eight studies, *n* = 1,342) [47, 48, 50, 52, 54, 55, 57, 59] and its activation fragment C3a (seven studies, *n* = 654) [24, 47–51, 58], followed by C4 (three studies, *n* = 90) [48, 50, 57], C4a (two studies, *n* = 56) [48, 51], C5 (three studies) [26, 47, 48], and C5a (two studies) [47, 48]. Several studies additionally quantified terminal complement complex (MAC) [26, 27, 47, 55], mannose-binding lectin (MBL) [55, 56], Factor B [25, 59], C1q [53], and other regulatory fragments (C3c [47], CH50 [48], soluble CD59 [26], C3dg [27], C3bc [27], C4c [27] and C4d [27]) (see Supplemental Table 1).

**Fig. 2:**
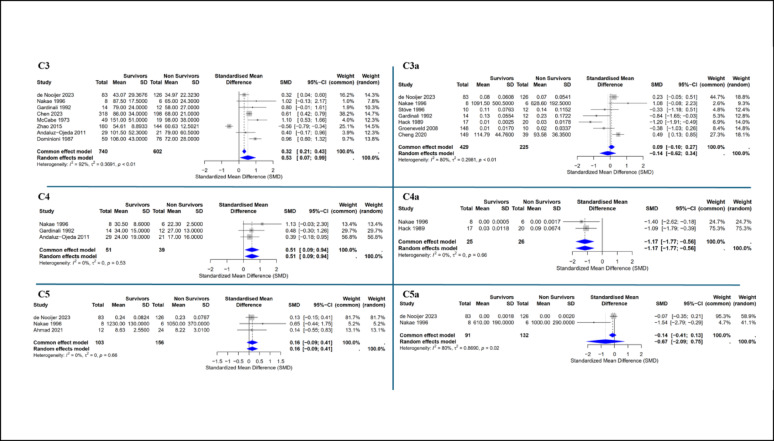
Meta-analysis of complement proteins in patients with sepsis. Forest plots comparing the levels of different complement proteins between survivors and non-survivors in the ICU. Each individual study is represented by a square, with its size proportional to the study's weight in the meta-analysis. Horizontal bars indicate 95% confidence intervals. The blue diamond represents the pooled effect estimate from the random-effects model. Heterogeneity values (I²) are provided for each analysis

Pooled analyses showed that C3 (SMD 0.53, 95% CI 0.07–0.99) and C4 (SMD 0.51, 95% CI 0.09–0.94) were significantly higher in survivors, consistent with complement depletion in non-survivors. The activation product C4a was significantly lower in survivors (SMD − 1.17, 95% CI − 1.77 to − 0.56), whereas C3a, C5, and C5a showed similar, non-significant trends toward higher levels in non-survivors. MAC concentrations did not differ between groups (Fig. 2; Supplemental Fig. 2).

#### 1b. Immunoglobulins

Thirteen studies reported on immunoglobulins [19–23, 57, 60–66], encompassing 2,594 patients (overall mortality 24%), reported circulating immunoglobulin levels. Among these, IgG (11 studies) [10, 20–23, 57, 60–63, 65, 66], IgM (12 studies) [19, 20, 22, 23, 57, 60–66], and IgA (9 studies) [20, 22, 23, 57, 60–63, 65] were the most frequently measured, with four studies [20, 60, 63, 65] reporting IgG subclasses and one study [63] measuring IgA subclasses (see Supplemental Table 2).

**Fig. 3:**
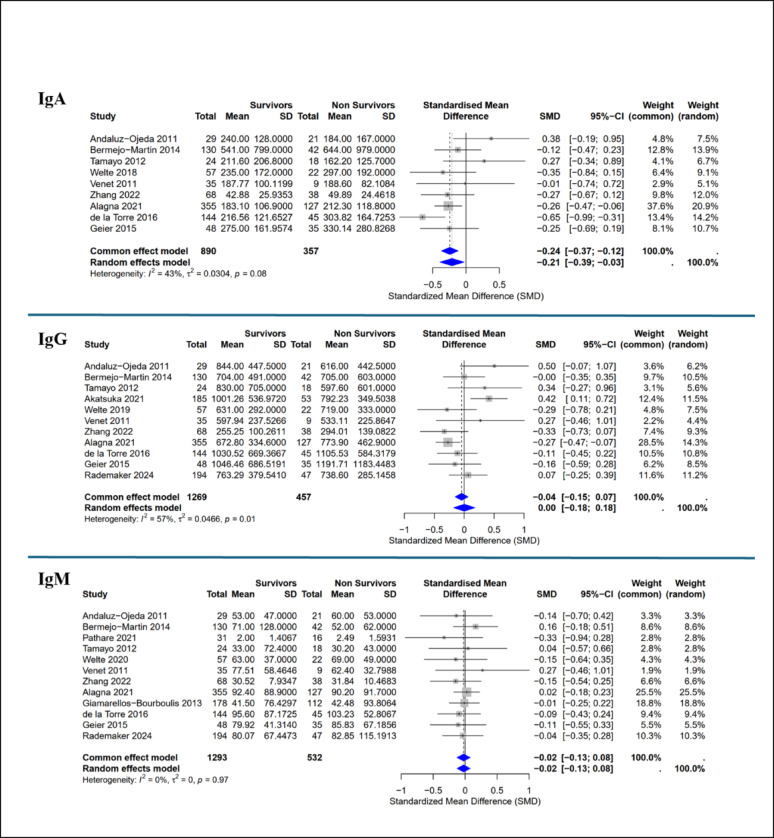
Meta-analysis of immunoglobulins in patients with sepsis. Forest plots comparing the levels of different immunoglobulin proteins between survivors and non-survivors in the ICU. Each individual study is represented by a square, with its size proportional to the study's weight in the meta-analysis. Horizontal bars indicate 95% confidence intervals. The blue diamond represents the pooled effect estimate from the random-effects model. Heterogeneity values (I²) are provided for each analysis

Pooled analyses revealed no significant differences in total IgG (SMD 0.00, 95% CI − 0.18 to 0.18) or IgM (SMD − 0.02, 95% CI − 0.13 to 0.08) between survivors and non-survivors. In contrast, IgA levels were modestly higher in non-survivors (SMD − 0.21, 95% CI − 0.39 to − 0.03) (Fig. 3).

Among IgG subclasses, IgG1, IgG3, and IgG4 did not differ significantly, whereas IgG2 tended to be higher in survivors, though not reaching statistical significance (SMD 0.25, 95% CI − 0.10 to 0.60; Supplemental Fig. 3).

#### 1c. Antimicrobial peptides

Seven studies [15–18, 67–69], comprising 1,290 patients with an overall 34% mortality, evaluated circulating antimicrobial peptides. All studies measured heparin-binding protein (HBP), while a single study additionally assessed α-defensins, bactericidal/permeability-increasing protein (BPI), lactoferrin, and LL-37 [67] (see Supplemental Table 3).

Pooled analysis showed that HBP levels tended to be lower in survivors compared with non-survivors (SMD − 0.44, 95% CI − 0.97 to 0.10; I² = 93%), although this did not reach statistical significance (Supplemental Fig. 1). Other peptides including defensins, BPI, lactoferrin, and LL-37, showed no significant differences between groups (Supplemental Fig. 4).

### Risk of bias analysis

The risk of bias for all 36 included studies in the meta-analysis was assessed using the NOS. The median NOS score was 7 (IQR 7–7.5). Overall, 32 studies (89%) were rated high quality (low risk of bias) and 4 (11%) moderate quality, with none rated low. Domain-level medians were Selection 3/4, Comparability 2/2, and Outcome 2/3 (Supplemental Table 6).

### Sensitivity analysis – Studies restricted to early sampling (≤ 24 h ICU admission)

When restricted to studies measuring proteins within the first 24 h of ICU admission (Supplemental Fig. 9), survivors had higher C3 and a similar trend for C4, while C3a and IgA were lower. No differences were observed for IgM or IgG.

### Sensitivity analysis – Pooled analysis including MIMIC-IV dataset

We performed a sensitivity analysis including the MIMIC-IV database, which reported protein measurements within the first 24 h of ICU admission (Supplemental Fig. 10). The inclusion of the MIMIC-IV cohort substantially increased the sample size for each protein. Specifically, MIMIC contributed 835 additional patients with IgA measurements, 1,023 with IgG, 767 with IgM, 323 with C3, and 336 with C4. Results were consistent with the main findings: survivors had higher C3 and C4, and lower IgA. IgM and IgG remained unchanged.

### Secondary analysis – MIMIC-IV database

We conducted a secondary analysis using the MIMIC-IV database (*n* = 2,452; mortality 9.1%), focusing on complement and immunoglobulin levels measured from admission to day 5 (Supplementary Table 4). Protein concentrations were dichotomized into early (days 0–1) and later (days 2–5) sampling (Supplemental Fig. 5). Data were available for 616 patients with C3, 630 with C4, 1,576 with IgA, 1,869 with IgG, and 1,443 with IgM.

IgA and IgM were significantly lower in survivors on days 0–1 but not on days 2–5; within-group analyses showed rising levels in survivors and declining levels in non-survivors. IgG did not differ at admission but was higher in survivors on days 2–5, increasing over time in survivors and decreasing in non-survivors. C3 was consistently higher in survivors at both time points, whereas C4 was higher only on days 2–5; both remained stable over time within groups.

### Secondary analysis – Proteomic data

To complement our meta-analysis, we analysed datasets from three published proteomic or glycoproteomic studies: Mi et al. [12], Langley et al. [14], and De Coux et al. [13] (Supplemental Table 5). Individual datasets quantified between 234 to over 1,000 plasma proteins, with up to 56 proteins classified as humoral immunity proteins (including complement components, immunoglobulins, and antimicrobial peptides).

Secondary analysis of the dataset from Mi et al. (1,189 ICU patients, 17% mortality) [12] showed early reductions in CFB, C3, and C4B in both survivors and non-survivors, with increases in C9, C1S, and C8G within the first 72 h of ICU Admission (Supplemental Fig. 6).

In the study by Langley et al. (121 septic patients in the emergency department, 36% mortality) [14], complement proteins and their regulators were higher in survivors at ICU admission, but declined sharply within the first 24 h, with a greater magnitude of reduction in non-survivors (Supplemental Fig. 7).

In the dataset from De Coux et al. (20 ICU patients, 50% mortality) [13], non-survivors had lower overall complement levels but increased terminal cascade proteins (C7, C9), whereas survivors showed higher levels of regulatory factors (Factor H, Factor I, C1q) and immunoglobulin-related proteins (J chain, polymeric Ig receptor, IgG Fc-binding protein) (Supplemental Fig. 8).

## Discussion

In this systematic review and meta-analysis of 36 studies involving more than 6,000 patients, we demonstrate that circulating levels of complement proteins C3 and C4 are consistently lower in sepsis non-survivors compared to survivors, whereas C4a, a cleavage product of complement activation, is markedly elevated. These associations remained robust in sensitivity and secondary analyses using both the MIMIC-IV clinical dataset [10] and three independent proteomic studies [12–14].

Complement plays a central role in pathogen clearance, but its overactivation can drive endothelial injury, coagulopathy, and multiorgan failure. The lower levels of C3 and C4 observed in non-survivors likely reflect consumption due to uncontrolled activation and/or insufficient synthesis under overwhelming infection [70]. Elevated C4a, and trends toward increased C3a, C5a, and soluble membrane attack complex (sC5b-9), reinforce the concept of maladaptive complement activation in sepsis non-survivors. These findings are consistent with prior observational studies showing that reduced circulating complement activity, as a surrogate for depletion, is associated with poor outcomes [24, 51, 55]. Collectively, our results provide integrative evidence that complement consumption is not merely an epiphenomenon of severe infection, but a reproducible biomarker of mortality risk across multiple independent datasets.

### Immunoglobulin dynamics and subclasses

Our pooled analysis did not identify significant baseline differences in total IgG or IgM between survivors and non-survivors. However, MIMIC-IV revealed divergent temporal trajectories: immunoglobulin levels increased over the first five days in survivors but decreased in non-survivors. This emphasizes the importance of immune dynamics, as static measurements may obscure prognostically relevant trajectories [66].

Among IgG subclasses, IgG2 levels were higher in survivors, although not statistically significant. This finding aligns with the known protective role of IgG2 against encapsulated bacteria such as *Streptococcus pneumoniae* and *Haemophilus influenzae* [71, 72]. In contrast, other subclasses (IgG1, IgG3, IgG4) showed no consistent associations. Although limited by small sample sizes, these results suggest that IgG2 deficiency may contribute to mortality risk.

IgM, despite its central role in early recognition and complement activation [73], did not differ significantly between groups in our pooled analyses. Prior studies linking low IgM to poor outcomes [19, 64], and meta-analyses suggesting IgM-enriched IVIg reduces mortality more effectively than standard IVIg [74], underscore the need for stratified clinical trials. The post-hoc CIGMA trial analysis showing benefits of trimodulin in patients with high CRP, low lymphocyte counts, and low IgM further supports personalized immunoglobulin therapy [75]. One previous analysis showed that the over-time kinetics of IgM were defective in non-survivors compared with survivors, whereas day-1 blood levels were similar [64]. This probably suggests that the rate of consumption of IgM is more important than the actual baseline levels.

Serum IgA levels were significantly higher in non-survivors. While predominantly a mucosal antibody, elevated systemic IgA may reflect epithelial barrier leakage or dysregulated systemic production [22, 76]. The paucity of subclass-specific data is notable, as IgA1 and IgA2 differ in glycosylation and function [77]. The pro-inflammatory properties of IgA2 on neutrophils and macrophages suggest that subclass-specific analyses may help determine whether IgA elevation is adaptive or pathogenic.

### Antimicrobial peptides

Heparin-binding protein (HBP) is secreted by activated neutrophils and influences vascular permeability and antimicrobial defense. Although our meta-analysis found no significant differences in HBP between survivors and non-survivors, prior studies have linked elevated HBP to circulatory failure, shock, and acute kidney injury [34, 69]. The lack of therapeutic development in this field is striking; recombinant bactericidal/permeability-increasing protein (rBPI21) showed a mortality reduction trend in pediatric meningococcal sepsis, though underpowered [78]. Our results suggest that while AMPs may hold promise as biomarkers, their therapeutic role remains unproven.

### Translational implications

Our findings underscore the translational potential of humoral immunity proteins both as accessible biomarkers for risk stratification and as therapeutic targets. Sepsis can be characterised by distinct sub-phenotypes, defined by distinct inflammatory, metabolic, and immune trajectories based on clinical parameters, and proteomic and transcriptomic profiles. Retrospective analyses suggest distinct biological processes as potential drivers of sepsis sub-phenotypes, which allows the identification of homogeneous cohort of patients likely to benefit from therapeutic intervention [79–81].

The inclusion of humoral immunity proteins to define sepsis sub-phenotypes may allow identification of patients most likely to benefit from therapeutic intervention with immunoglobulin supplementation or complement pathway modulation. While inhibition of complement activation among patients with a hyperinflammatory phenotype may mitigate excessive inflammation, endothelial injury, and coagulopathy, complement supplementation in the hypoinflammatory phase may restore immune competence in patients with complement depletion. Similarly, IgM- enriched preparations (such as trimodulin) may confer survival benefits in subgroups with low baseline IgM levels or increased risk of death.

We found lower levels of C3 and C4 (suggesting consumption) were associated with mortality. However, recent data suggest that in very severe sepsis/septic shock complement levels may be universally altered and no longer discriminate outcome [47]. Nevertheless, the discovery of subphenotypes, and the possibility to link those phenotypes to easily measurable biomarkers can help to select patients that may benefit from single or combined therapies. With recent advances in diagnostics capable of measuring circulating proteins and host gene-expression, it is becoming increasingly feasible to conduct precision-medicine clinical trials based on subphenotypes.

### Limitations and future directions

Despite the comprehensive analyses conducted, we acknowledge limitations to our study. Substantial heterogeneity exists among the included studies in patient populations, sepsis definitions, timing of sampling, and assay methodologies. Additionally, lack of data on patient-level covariates preclude adjustment for potential confounders such as comorbidities, illness severity, and infection source. We accounted for heterogeneity between studies by using a random-effects model in our meta-analyses. Most studies report single baseline measurements of humoral immune proteins. We addressed this by evaluating temporal data from the MIMIC-IV and proteomic cohorts. Publication bias cannot be excluded, as studies with negative or inconclusive results are less likely to be published. The observational nature of studies limits causal inference.

In this study, analyses were exploratory and hypothesis-generating, aiming to identify broad patterns. Although proteomic analyses were not corrected for multiple comparisons, proteins with overlapping functional pathways change in parallel. This suggests that the differences in functional pathways between sepsis survivors and non-survivors may be underpinned by a common biological pathway, rather than representing chance findings.

Future research should focus on prospective multicentre studies incorporating calibrated assays, predefined multiple sampling time points, and integration of humoral immunity proteins into sepsis sub-phenotyping analyses. Mechanistic investigations are needed to clarify how complement protein and immunoglobulin trajectories can be modulated to improve immune function and patient outcomes.

## Conclusion

Lower circulating levels of classical complement proteins are consistent features of sepsis non-survivors. These findings are consistent across meta-analysis of observational studies, a large clinical database, and proteomic studies, highlight humoral immunity is a relatively neglected determinant of outcome in sepsis. Complement and immunoglobulin profiles may serve as biomarkers for risk stratification and as targets for immunotherapy. Prospective studies integrating standardized complement assays, immunoglobulin subclass profiling, and stratified immunoglobulin therapy are warranted to translate these insights into therapeutic interventions for sepsis.

## Supplementary Information

Supplementary Material 1

## Data Availability

The datasets generated and/or analyzed during the current study are available from the corresponding author upon reasonable request.

## Abbreviations

AMP: Antimicrobial peptide
BPI: Bactericidal/permeability-increasing protein
CI: Confidence interval
HBP: Heparin-binding protein
ICU: Intensive care unit
Ig: Immunoglobulin
IgA: Immunoglobulin A
IgG: Immunoglobulin G
IgM: Immunoglobulin M
IVIg: Intravenous immunoglobulin
MAC: Membrane attack complex
MBL: Mannose-binding lectin
NOS: Newcastle–Ottawa Scale
PRISMA: Preferred Reporting Items for Systematic Reviews and Meta-Analyses
PROSPERO: International Prospective Register of Systematic Reviews
SMD: Standardized mean difference
