# Supplementary material for: Systematic review and meta-analysis of humoral immunity proteins and mortality in sepsis
Source: Crit Care. 2025 Dec 22;30:41. doi: 10.1186/s13054-025-05758-0 (PMC12837105; doi:10.1186/s13054-025-05758-0)
Supplement: Supplementary file 1 — Supplementary Material 1 [file 13054_2025_5758_MOESM1_ESM.docx]

Studies investigating complement proteins

| Parameter | de Nooijer et al. | Nakae et al. | Gardinali et al. | Stöve et al | Chen et al. | Hack et al. | McCabe et al. | Li et al | Zhao et al. | Zogheib et al | Dionigi et al | Ahmad et al | Groeneveld et al | Andaluz-Ojeda et al. | Dominioni et al. | Cheng et al | Hedetoft et al |
| --- | --- | --- | --- | --- | --- | --- | --- | --- | --- | --- | --- | --- | --- | --- | --- | --- | --- |
| Sample size | 209 | 14 | 26 | 22 | 516 | 37 | 68 | 132 | 324 | 27 | 38 | 36 | 158 | 50 | 135 | 188 | 242 |
| Mortality | 60% (126) | 42% (6) | 46% (12) | 54% (12) | 38%(198) | 57% (20) | 27% (19) | 33% (43) | 44% (144) | 37% (10) | 47% (18) | 66% (24) | 6% (10) | 42% (21) | 56%(76) | 20% (39) | 17% (41) |
| Proteins measured | C3 C3a C3c C5 C5a TCC | CH50 C3 C3a C4 C4a C5 C5a | C3 C4 C3a | C3a | C3 | C3a  C4a | C3 | C1q | C3 MAC MBL | MBL | FB | C5 MAC sCD59 | C3a | C3 C4 | FB C3 | C3a | C4c C4d C3bc C3dg MAC |
| Recruitment period | December 2017 and December 2019 | Not mentioned | Not mentioned | Not mentioned | June 2017 October 2022 | Not mentioned | Not mentioned | July 2020 to March 2021 | Not mentioned | Not mentioned | 1982-1983 | June 2020 and January 2021 | Not mentioned | January 2010 to January 2011 | October 1982 and October 1985 | Not mentioned | February 2013 and March 2017 |
| Setting | Multicentric | Single center | Single center | Single center | Single center | Single center | Single center | Single center | Single center | Single center | Single center | Single center | Single center | Single center | Multicenter | Multicenter | Multicenter |
| Location | Greece | Japan | Italy | Germany | China | Netherlands | USA | China | China | France | Italy | Jordan | Netherlands | Spain | Italy | USA | Denmark |
| Sepsis type | Community acquired and HAI | Sepsis (various causes) | Severe sepsis (bacterial) | Sepsis, severe sepsis | Sepsis | Sepsis | Sepsis | Sepsis, severe sepsis | Severe sepsis, septic shock | H1N1 sepsis | Septic surgical patients | Sepsis | Sepsis | Severe sepsis, septic shock | Septic surgical patients, | Sepsis | Necrotsing Soft Tissue infection |
| Primary aim to look at complement | Yes | Yes | Yes | Yes | Yes | Yes | Yes | Yes | Yes | Yes | Yes | Yes | Yes | Yes | Yes | Yes | Yes |
| Complement measurement method | ELISA | Double Antibody RIA | Radial Immunodiffusion | ABICAP | Immunoturbidimetry | RIA | Radial Immunodiffusion | Immunoturbidimetric method | ELISA | ELISA | Nephelometry | ELISA | RIA | DadeBehringBNII | Nephelometry | Western Blot | ELISA |
| Measurement time point | within 24 h after sepsis diagnosis | At diagnosis | At ICU admission | At ICU admission | Admission in the hospital | Admission to ICU | At admission | At admission | ED arrival | within 24 h of ICU admission | On the day of diagnosis | Within 48h of admission | ICU admission | Day 1 after ICU | At hospital admission | At hospital admission | At hospital admission |
| Mortality reported | 28 days | hospital | hospital | hospital | 28 days | In ICU | In hospital | 28 days | Yes | 30-days | In ICU | ICU stay | 28 days | In ICU | In hospital | 28 days | 30-days |
| Used consensus definition of sepsis | Yes | Yes | Yes | Yes | Yes | Yes | No | Yes | Yes | Yes | Yes | Yes | Yes | Yes | Yes | Yes | Yes |
| Source of data for scoring | Full text and author contact | Full text | Full text | Full text | Full text | Full text | Full text | Full text | Full text | Full text | Full text | Full text | Full text | Full text and author contact | Full text | Full text | Full text and author contact |
| Age mean(SD) | 76 [65–84] | 52.9 ± 16.5 | 52 | 45 | - | - | - | 56.88 ± 14.2 | 67.9 ± 13.6 | 56 ± 29 |  | 63.1 ± 15.7 | 64 (17—97) | 68.5 (19.2) |  |  | 62 (51–70) |
| Sex male n(%) | 119 (57) | 8 (57) | 20 (77) | - 14(64) | 275 (53) | - | - | 78(60) | 114 ( 63.7%) | - |  | 16 (44%) | 81 (51) | 32 (64%) |  |  | 144 (60) |
| Culture positive  GP   GN   Other  Culture Negative | - |  | 6  19  1 | 10  5  7 |  | 11  23 | 68 | 13  47  72  58 |  |  | GP 67%  GN 26% |  |  | 36(72%)  14(28%)  19(38%)  3(6%)  14(28%) |  |  | 222(92%)  50(21%) |
| Infection type | Nosocomial and community | Community | Community | Community | Community | Community | Community | Community | Community | Community | Nosocomial and community | Community | Community | Community | Community | Community | Community |
| Infection site   Lung   Blood   UT  Abdomen  Other  Site unknown | 161  34    13 | 3     3  8 | 3      14 (0)  9 (20)  1  1 | 10  3    3  6 | 60 (Lung)      336  Abdo 132  120 (Other) | - | 68 | 40   36   39  40  17 | 110    15  54 | 27 | 100% Abdomen |  | 71  4  29  23 | 23(46%)  NA  9(18%)  5(10%)  NA |  |  | Necrotising soft tissue infection |
| ICU LOS | - | - |  |  |  |  |  |  |  | 22 [IQR 23] | - | 11.8 ± 10.7 |  | NA | NA | NA | NA |
| Hospital LOS | - | - |  |  |  |  |  |  |  |  | - | 15.9 ± 10.5 |  | NA | NA | NA | NA |
| SOFA | 11 (9-14) |  |  |  | 8 +-1 |  |  | 5(3,8) |  | 12 [IQR 5] |  | 8.3 ± 4.8 | - | 8.0 (4.0) | NA | NA | 8 (6–10) |
|  |  |  |  |  |  |  |  |  |  |  |  |  |  |  |  |  |  |

**Supplemental Table 1. Characteristics of Studies Investigating Complement Proteins Included in the Meta-Analysis**

This table summarizes the characteristics of studies evaluating complement proteins in septic patients, including sample size, mortality, measured proteins, methods of measurement, timing of sampling, and patient populations.
**Abbreviations**:
C3, C4, C5 = Complement components;
C1q = Complement component 1q;
MAC = Membrane attack complex;
MBL = Mannose-binding lectin;
FB = Factor B;
sCD59 = soluble CD59;
CH50 = Total hemolytic complement activity;
TCC = Terminal complement complex;
ELISA = Enzyme-linked immunosorbent assay;
RIA = Radioimmunoassay;
LOS = Length of stay;
ICU = Intensive Care Unit;
ED = Emergency Department;
GP = Gram-positive;
GN = Gram-negative;
UT = Urinary tract.

Studies investigating Immunoglobulins

|  | Andaluz-Ojeda 2011 | Bermejo-Martin 2014 | Pathare 2021 | Tamayo 2012 | Akatsuka 2021 | Welte 2018 | Venet 2011 | Zhang 2022 | Alagna 2021 | Giamarellos 2013 | De la Torre 2016 | Geier 2015 | Rademaker 2024 |
| --- | --- | --- | --- | --- | --- | --- | --- | --- | --- | --- | --- | --- | --- |
| Sample size | 50 | 172 | 47 | 42 | 238 | 79 | 44 | 106 | 956 | 290 | 189 | 83 | 255 |
| Mortality | 42% (21) | 24% (42) | 34% (16) | 42% (18) | 22% (53) | 27% (22) | 20% (9) | 35% (38) | 13% (127) | 38% (112) | 23% (45) | 42% (35) | 19% (47) |
| Proteins measured | IgG, IgM, IgA | IgG1,G2,G3,G4, IgA,IgM, IgE | IgM | IgG IgG1 IgG2 IgG3 IgG4 IgM IgA | IgG | IgG, IgM, IgA | IgG IgA IgM | IgA1-2,IgG1-4,IgM | IgA,IgM,IgG | IgM | IgG1-4, IgA, IgM | IgG,IgM,IgA | IgG, IgM |
| Recruitment period | January 2010 to January 2011 | January 2012 to December 2012 | January 2011 to March 2014 | January to December 2011. | January 2013 to August 2018 | October 2011 to February 2015 |  | February 2017 to February 2019 | July 2008 to October 2013 | January 2010 to December 2010 | 01/2001-03/2012 | May 2012 until April 2013, | 01/2011-06/2024 |
| Setting | Single centre | Multicentric | Single centre | Single centre | Single centre | Multicentric | Single centre | Single centre | Multicentric | Multicentric | single centre | single centre | Multicentric |
| Location | Spain | Spain | Wales | Spain | Japan | Germany, Spain, UK | France | China | Italy | Greece | Spain | Germany | Netherlands |
| Sepsis type | Sepsis, severe sepsis, septic shock | Severe sepsis, septic shock | Sepsis | Septic shock | Sepsis | sCAP | Septic shock | Sepsis | Severe sepsis, septic shock | Sepsis, septic shock | CAP | Sepsis | CAP Sepsis-3 defined septic shock |
| Primary aim to look at Ig level | Yes | Yes | Yes | Yes | Yes | No | Yes | Yes | No | Yes | yes | Yes | Yes |
| Ig measurement method | Nephelometry | Luminex assay | ELISA | Multiplex Immunoglobulin Isotyping kit | Not reported | Not reported | nephelometry | Fluorescence assay | Turbidimetry | enzyme-linked immunosorbent assay | nephelometry | Turbidimetry | Turbidimetry |
| Measurement time point | Day 1, Day 3, Day 10 of admission | On diagnosis of sepsis | Within 12hrs of ICU admission | in the 24 first hours following diagnosis | On ICU admission | sCAP on IMV in ICU baseline day 1 n=79, and day 5, 7, 21 , in n=5-9 | days 1–2 | On enrollment | Day 1, 2, 7 after enrollment | Within 24hours of time of diagnosis | first contact and time of cap diagnosis | On arrival to ICU | Upon ICU admission |
| Mortality reported | Within 28 days | ICU | ICU mortality | Within 28 days | Within 28 days | Within 28 days | Within 28 days | Within 28 days | Within 90 days | Within 28 days | 30 day | ICU mortality | ICU mortality |
| Used consensus definition of sepsis | Yes | Yes | Yes | Yes | Yes | Yes | Yes | Yes | Yes | Yes | no | Yes | Yes |
| Source of data for scoring | Full text | Full text | Full text | Full text | Full text | Full text | Full text and author contact | Full text | Full text | Full text and author contact | Full text | Full text and author contact | Full text and author contact |
| Age mean(SD) | 68.5 (19.2) | 68.5 (19.0) | 63 (54-71) | 68.3 (14.4) | 65.4 | 65.5 (14.8) | 72 [62–80] | 78.31 (10.86) | 66.6 (14.7) | 70(16.9) | 60.1(17.4) | 62 ± 14 | 62 [48–70] |
| Sex male n(%) | 32 (64%) | 115 (66.9) | 23 (49) | 30 (71%) | 66.4% | 57 (72.2) | 39 (63) | 62 (58.5) | 561 (58.7) | 144(49.5) | 146(71.2) | 54 (65.1 %) | 159 (62) |
| Culture positive  GP   GN   Other  Culture Negative | Not reported 14 19 not reported | Not reported  39 58 11 | 28 15 13 0  16 |  |  | 34  12  6 (other)  33 (viral) | Bacilli Gram− 30 (48) Cocci Gram+ 28 (45) |  |  | unclear | not reported |  | *S. pneumoniae* 58(23%) or *H. influenzae*  29(11%) |
| Infection type community vs. nosocomial | Not mentioned | Not mentioned | Not reported | Community | Not reported | Community | community and nosocomial | Not reported | Not reported | Community and nosocomial | Community |  | Community |
| Infection site   Lung   Blood   UT  Abdomen  Other  Site unknown | 23 ? 9 5 | 57 9 26 60 20 | 15 ? 5 17 10 |  | 91 7 13 76 51 | 79 (Lung 100%) | Pulmonary 24 (39) Abdominal 27 (44) Others 11 (18) |  | 326 ? 89 329 212 | 115(40%)  46(16%)  81(28%)  49(17%)  0(0%) | lung 100% |  | lung 100% |
| ICU LOS |  | 8 (12.0) | 8 (4-17) | Survivors 35.0 (119.1) NS 18.4 (18.7) |  | 14.4 (5.82) | 18 [18–31] |  | Not reported for secondary analysis | NA | not reported |  | 10 [6–16] |
| Hospital LOS |  | 25 (25.0) | 27 (15-66) |  |  | 14.4 (5.82) |  | 6.66 (6.35) | Not reported for secondary analysis | NA | not reported |  | not reported |
| SOFA/APACHE II | SOFA - 8.0 (4.0) | APACHE II - 21 (10.0) | SOFA day 1- 15(13-17)  APACHE II 17 (14-21) | APACHE S 13.0 (5.6) NS 18.4 (3.5) | SOFA 8 (5-10)  APACHE 22 (18-26) | SOFA: 10.5 (3.8)  APACHE: 26.2 (8.3) | SAPS II at diagnosis of shock* 52 [42–64] SOFA Score* 10 [9–13] | 5.0 (2.56) | 8.9 (2.4) | 18.4±6.5  (apache) | not reported | 28.1 ± 8.3 (APACHE) | SOFA 7 [6–9] |

**Supplemental Table 2. Characteristics of Studies Investigating Immunoglobulins Included in the Meta-Analysis**

This table presents the clinical and methodological characteristics of studies assessing immunoglobulin levels in patients with sepsis, including study design, proteins evaluated, timing, and outcomes.
**Abbreviations**:
IgG, IgA, IgM = Immunoglobulin G, A, M;
IgG1–4 = IgG subclasses;
IgA1–2 = IgA subclasses;
CAP = Community-acquired pneumonia;
SAPS II = Simplified Acute Physiology Score II;
SOFA = Sequential Organ Failure Assessment;
APACHE II = Acute Physiology and Chronic Health Evaluation II;
LOS = Length of stay;
ICU = Intensive Care Unit;
GP = Gram-positive;
GN = Gram-negative;
UT = Urinary tract.

Studies investigating antimicrobial peptides and proteins

| Parameter | Ingrid 2010 | Xue 2023 | Tang 2023 | Bergquist 2020 | Dou 2022 | Katsaros 2022 | Tverring 2017 |
| --- | --- | --- | --- | --- | --- | --- | --- |
| Sample size | 31 | 146 | 51 | 248 | 206 | 139 | 511 |
| Mortality | 39% (12) | 44% (64) | 71% (38) | 7% (18) | 66% (137) | 19% (27) | 28% (145) |
| Proteins measured | BPI, HBP, alpha defensins, lactoferrin, ll-37, | HBP | HBP | HBP | HBP | HBP | HBP |
| Recruitment period | 23/03/2004-07/12/2007 | 07/2020-06/2022 | 01/2019-09/2022 | 01/02/2012-31/01/2013 | 08/2019-01/2020 | 26/09/2017-01/09/2018 | 01/09/2011-01/02/2012 |
| Setting | Single centre | Single centre | Single centre | Single centre | Single centre | Multicentre | Multicentre |
| Location | Sweden | China | China | Sweden | China | Greece | Finland |
| Sepsis type | Sepsis (severe sepsis, septic shock) | Septic shock | Sepsis | Sepsis | Sepsis | Sepsis | Severe sepsis, septic shock |
| Primary aim to look at AMP | Yes | Yes | Yes | Yes | yes | yes | yes |
| AMP measurement method | (sandwich) ELISA | immunofluorescence assay | Jet-iStar3000 automatic immune analyzer | Briefly, plates were coated with a mouse monoclonal antibody directed against HBP | elisa | fluorescence dry quantitative immunoassay | elisa |
| Measurement time point | Day of inclusion, day 4 | at admission and 72hrs later | 1st, 3rd, 5th day after admission | At admission, day 2 & day 3 | at admission, 24 hrs, 48hrs | within 1st hr of ed admission | at admission up to 2 hrs after admission |
| Mortality reported | 90 day | 28 days | 28 day | 30 day | 30 day | 72 hr, 28 day | 28 day |
| Used consensus definition of sepsis | Yes | Yes | Yes | Yes | Yes | Yes | No |
| Source of data for scoring | Full text | Full text | Full text | Full text | Full text | Full text | Full text |
| Age mean(SD) | Not reported (median) | 65,38 | 54.24 (16.24) | 73 (62-76) | Not reported (median) | 75.2(14.5) | not reported (median) |
| Sex male n(%) | 12(38.7) | 79(54.11) | 27(52.9) | 172 (61.87)  Sepsis group 54 (61) | 136(66.1) | 88(53) | 319 (62.43) |
| Culture positive  GP   GN   Other  Culture Negative | 51.6%, GP 10, GN 8, other 0 | not reported | Not reported | Not reported | not reported | Blood culture GN 7.8%  Urine culture 17% | cuture pos-22.30, gn- 9.98 |
| Infection type community vs. nosocomial | Not reported | not reported | not reported | Not reported | not reported | Community | community/nosocomial |
| Infection site   Lung   Blood   UT  Abdomen  Other  Site unknown | Not reported | lungs 65.7, blood 43.8 | 35.29 (Lung)  15.69 (UT)  21.57 (Other) | Lung 34%  Bacteraemia (unknown) 20%  UT 11%  Abdomen 18% | 87.38, 5.33, 5.33, 16.02, 3.88 | 93(56%)  4(2%)  38(23%)  21(13%) | 50.49  not reported  7.05  24.66  not reported |
| ICU LOS (mean) | not reported | 11,94 | Not reported | 3 (1-6) | not reported (median) | not reported | not reported |
| Hospital LOS | not reported | not reported | 8.32 (+/-2.63) days | Not reported | not reported (median) | not reported | not reported |
| SOFA | 3 (0-4) | 10,33 | 8.86 (+/-2.76) | 8 (6-11) | not reported (median) | 3.29(+/-1.92) | not reported (medianfor 601 patients) |

**Supplemental Table 3. Characteristics of Studies Investigating Antimicrobial Peptides (AMPs) Included in the Meta-Analysis**

Overview of studies evaluating antimicrobial peptides and proteins in sepsis, including recruitment details, measurement methods, timing, and clinical characteristics.
**Abbreviations**:
AMP = Antimicrobial peptide;
HBP = Heparin-binding protein;
BPI = Bactericidal/permeability-increasing protein;
LL-37 = Human cathelicidin;
ELISA = Enzyme-linked immunosorbent assay;
ICU = Intensive Care Unit;
ED = Emergency Department;
LOS = Length of stay;
GP = Gram-positive;
GN = Gram-negative;
UT = Urinary tract.

MIMIC database– Supplemental Data

|  | C3,  N = 616*^1^* | C4,  N = 630*^1^* | Immunoglobulin A,  N = 1,576*^1^* | Immunoglobulin G,  N = 1,869*^1^* | Immunoglobulin M,  N = 1,443*^1^* |
| --- | --- | --- | --- | --- | --- |
| Time | | | | | |
| 0-1 days | 323 (52%) | 336 (53%) | 835 (53%) | 1,023 (55%) | 767 (53%) |
| 2-5 days | 293 (48%) | 294 (47%) | 741 (47%) | 846 (45%) | 676 (47%) |
| Gender | | | | | |
| F | 331 (54%) | 337 (53%) | 716 (45%) | 818 (44%) | 654 (45%) |
| M | 285 (46%) | 293 (47%) | 860 (55%) | 1,051 (56%) | 789 (55%) |
| Survival | | | | | |
| Alive | 551 (89%) | 566 (90%) | 1,434 (91%) | 1,691 (90%) | 1,309 (91%) |
| Dead | 65 (11%) | 64 (10%) | 142 (9.0%) | 178 (9.5%) | 134 (9.3%) |
| Age | 53 (42, 65) | 53 (42, 65) | 61 (51, 71) | 61 (51, 70) | 61 (51, 71) |
| *^1^ n (%); Median (IQR)* | | | | | |

**Supplemental Table 4. Characteristics of Patients from the MIMIC-IV Cohort Included in the Meta-Analysis**

This table details the patient characteristics from the MIMIC-IV ICU database cohort included in the meta-analysis, stratified by complement and immunoglobulin protein measurements.
**Abbreviations**:
MIMIC-IV = Medical Information Mart for Intensive Care, version IV;
ICU = Intensive Care Unit;
IgA, IgG, IgM = Immunoglobulin A, G, M;
C3, C4 = Complement components;
IQR = Interquartile range.

OMICS Studies – Supplemental Data

|  | Mi [68] | Langley [70] | De Coux [69] |
| --- | --- | --- | --- |
| Timepoints (days) | D1, D3, D5 | D1, D2 | D1 |
| Patient numbers | 1189 | 121 | 20 |
| Mortality (%) | 17% | 26% | 50% |
| Number of proteins analysed | 63 (+) | 163 | 500 |
| Cohort | ICU | ED | ICU |

**Supplemental Table 5. Characteristics of OMICS-Based Studies Included in the Meta-Analysis**

Details of included proteomic studies analyzing multiple humoral immunity proteins in sepsis, with cohort characteristics, sample timing, and mortality rates.
**Abbreviations**:
ED = Emergency Department;
ICU = Intensive Care Unit;
D1, D2, D3, D5 = Day 1, 2, 3, and 5.

Newcastle–Ottawa Scale (NOS)

| Study | Selection (0–4) | Comparability (0–2) | Outcome (0–3) | Total NOS (0–9) | Study Quality |
| --- | --- | --- | --- | --- | --- |
| Serum levels of immunoglobulins and severity of community-acquired pneumonia | 3 | 2 | 2 | 7 | High quality |
| Dynamic changes in heparin-binding protein as a prognostic biomarker for 30-day mortality in sepsis patients in the intensive care | 3 | 2 | 2 | 7 | High quality |
| TNFR1, TNFR2, neutrophil gelatinase-associated lipocalin and heparin binding protein in identifying sepsis and predicting outcome | 3 | 2 | 2 | 7 | High quality |
| Kinetics of circulating immunoglobulin M in sepsis relationship with final outcome | 3 | 2 | 2 | 7 | High quality |
| Longitudinal assessment of immunoglobulin response and disease progression in critically ill patients with community acquired …. | 3 | 2 | 2 | 7 | High quality |
| Influence of the serum levels of immunoglobulins on clinical outcomes in medical intensive-care patients | 3 | 2 | 2 | 7 | High quality |
| Heparin Binding Protein for the Early Diagnosis and Prognosis of Sepsis in the Emergency Department The Prompt Multicenter Study | 3 | 2 | 2 | 7 | High quality |
| The Predictive Value of Heparin-Binding Protein and D-Dimer in Patients with Sepsis | 3 | 2 | 2 | 7 | High quality |
| Immunoglobulins IgG1, IgM and IgA a synergistic team influencing survival in sepsis | 3 | 2 | 2 | 7 | High quality |
| Higher levels of IgA and IgG at sepsis onset are associated with higher mortality results from the Albumin Italian Outcome Sepsis | 3 | 2 | 2 | 7 | High quality |
| Low immunoglobulin G level is associated with poor outcomes in patients with sepsis and septic shock | 3 | 2 | 2 | 7 | High quality |
| Changes in Heparin-Binding Protein, Procalcitonin, and C-Reactive Protein Within the First 72 Hours Predict 28-Day Mortality in Pa | 3 | 2 | 2 | 7 | High quality |
| Heparin-binding protein (HBP) improves prediction of sepsis-related acute kidney injury | 3 | 2 | 2 | 7 | High quality |
| Plasma IgM Levels Differentiate between Survivors and Non-Survivors of Culture-Positive and Culture-Negative Sepsis and SIRS …. | 3 | 2 | 2 | 7 | High quality |
| Elevated plasma levels of heparin-binding protein in intensive care unit patients with severe sepsis and septic shock | 3 | 2 | 2 | 7 | High quality |
| Prediction of short-term mortality in elderly patients with sepsis using immunoglobulin G2 An observational study | 3 | 2 | 2 | 7 | High quality |
| Serum Complement Levels in Bacteremia Due to Gram-Negative Organisms | 2 | 1 | 2 | 5 | Moderate quality |
| The Prognostic Performance of the Complement System in Septic Patients in Emergency Department A Cohort Study | 3 | 2 | 2 | 7 | High quality |
| The role of the innate immune response in hospital- versus community-acquired infection in febrile medical patients | 4 | 2 | 2 | 8 | High quality |
| Use of cardiac troponin I, lactic acid, procalcitonin, and serum complement C3 as prognostic indicators in patients with sepsis… | 4 | 2 | 2 | 8 | High quality |
| Sepsis Score and Complement Factor B for Monitoring Severely Septic Surgical Patients and for Predicting Their Survival | 4 | 2 | 2 | 8 | High quality |
| Sepsis Score and Acute-Phase Protein Response as Predictors of Outcome in Septic Surgical Patients | 3 | 2 | 2 | 7 | High quality |
| Serum C1q Levels Have Prognostic Value for Sepsis and are Related to the Severity of Sepsis and Organ Damage | 3 | 2 | 2 | 7 | High quality |
| Efficacy and safety of trimodulin, a novel polyclonal antibody preparation, in patients with severe community-acquired pneumonia … | 4 | 2 | 2 | 8 | High quality |
| Assessment of plasmatic immunoglobulin G, A and M levels in septic shock patients | 3 | 2 | 2 | 7 | High quality |
| Beneficial role of endogenous immunoglobulin subclasses and isotypes in septic shock | 3 | 2 | 2 | 7 | High quality |
| Prospective Observational Study on the Association Between Serum Mannose-Binding Lectin Levels and Severe Outcome in Critically… | 3 | 2 | 2 | 7 | High quality |
| Circulating Complement C3-Alpha Chain Levels Predict Survival of Septic Shock Patients | 4 | 2 | 2 | 8 | High quality |
| Circulating complement proteins in patients with sepsis or systemic inflammatory response syndrome. | 4 | 2 | 2 | 8 | High quality |
| Chronological changes in the complement system in sepsis | 2 | 1 | 2 | 5 | Moderate quality |
| Elevated plasma levels of the anaphylatoxins C3a and C4a are associated with a fatal outcome in sepsis | 2 | 1 | 2 | 5 | Moderate quality |
| Complement Activation and Polymorphonuclear Neutrophil Leukocyte Elastase in Sepsis Correlation With Severity of Disease | 3 | 1 | 2 | 6 | Moderate quality |
| Complement Terminal Pathway Activation is Associated with Organ Failure in Sepsis Patients | 3 | 2 | 2 | 7 | High quality |
| Early natural killer cell counts in blood predict mortality in severe sepsis | 3 | 2 | 2 | 7 | High quality |
| Complement activation in severely ill patients with sepsis no relationship with inflammation and disease severity | 4 | 1 | 2 | 7 | High quality |
| Increase in the Complement Activation Product C4d and the Terminal Complement Complex sC5b-9 Is Associated with Disease Severity … | 4 | 2 | 2 | 8 | High quality |

**Supplemental Table 6.** **Newcastle–Ottawa Scale (NOS) assessment of included studies.**

Scores are shown for each domain (Selection, Comparability, Outcome) with total score (0–9) and overall study quality (High, Moderate, Low).

# Supplemental figures

| 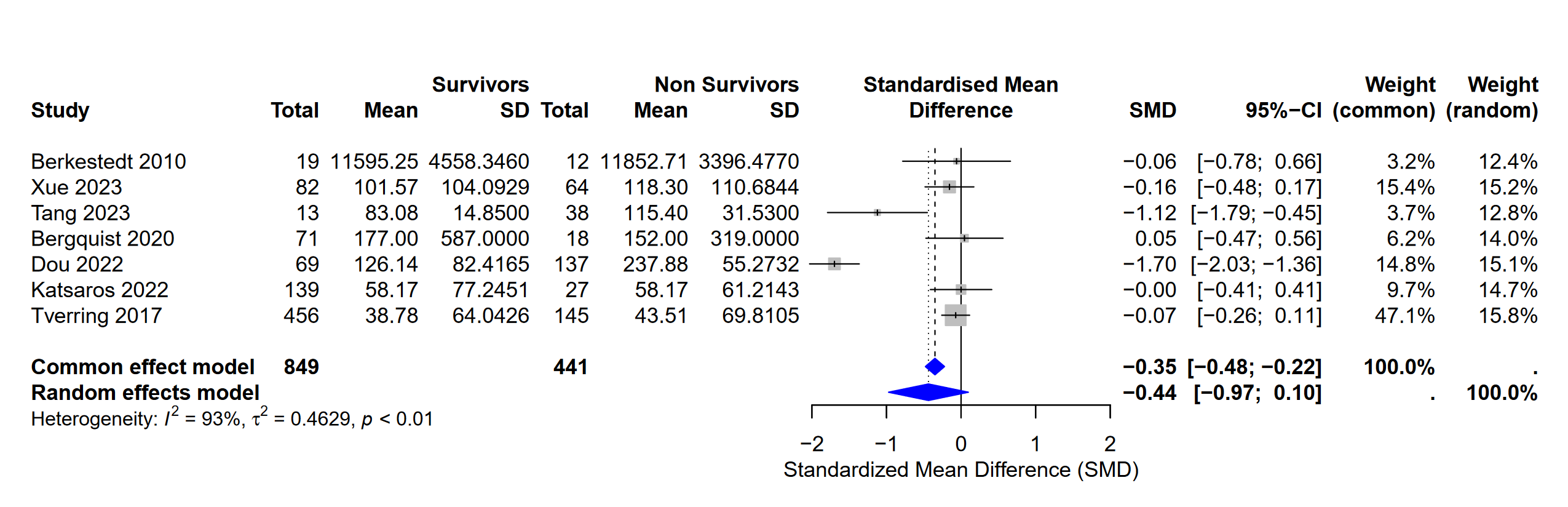  Supplemental Figure 1: **Meta-analysis of Heparin Binding Protein (HBP) in patients with sepsis.** *Forest plots comparing the levels of different HBPt proteins between survivors and non-survivors in the ICU. Each individual study is represented by a square, with its size proportional to the study's weight in the meta-analysis. Horizontal bars indicate 95% confidence intervals. The blue diamond represents the pooled effect estimate from the random-effects model. Heterogeneity values (I²) are provided for each analysis.* |
| --- |

| 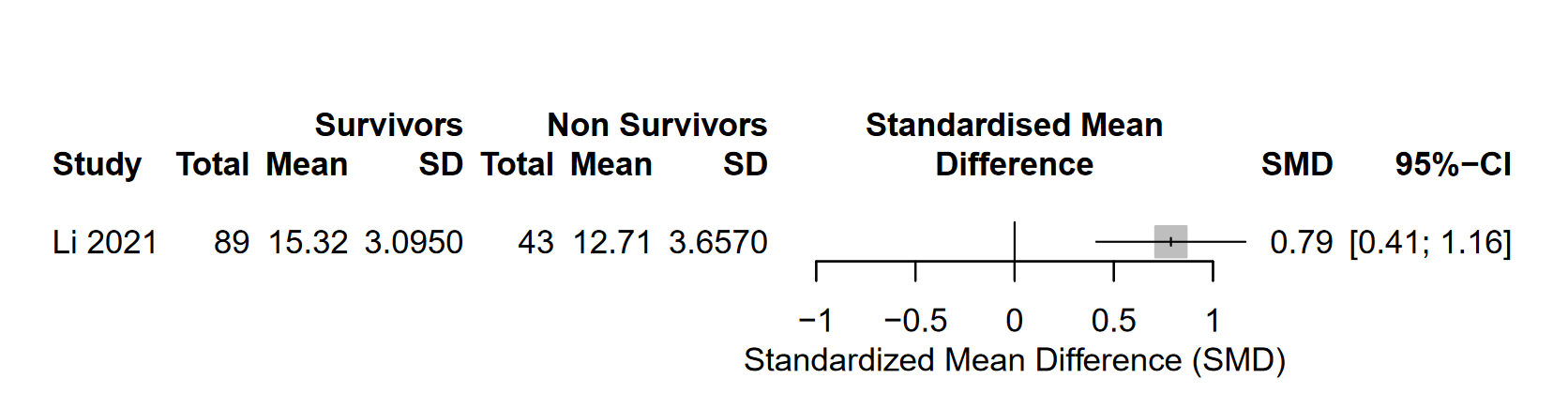  C1q  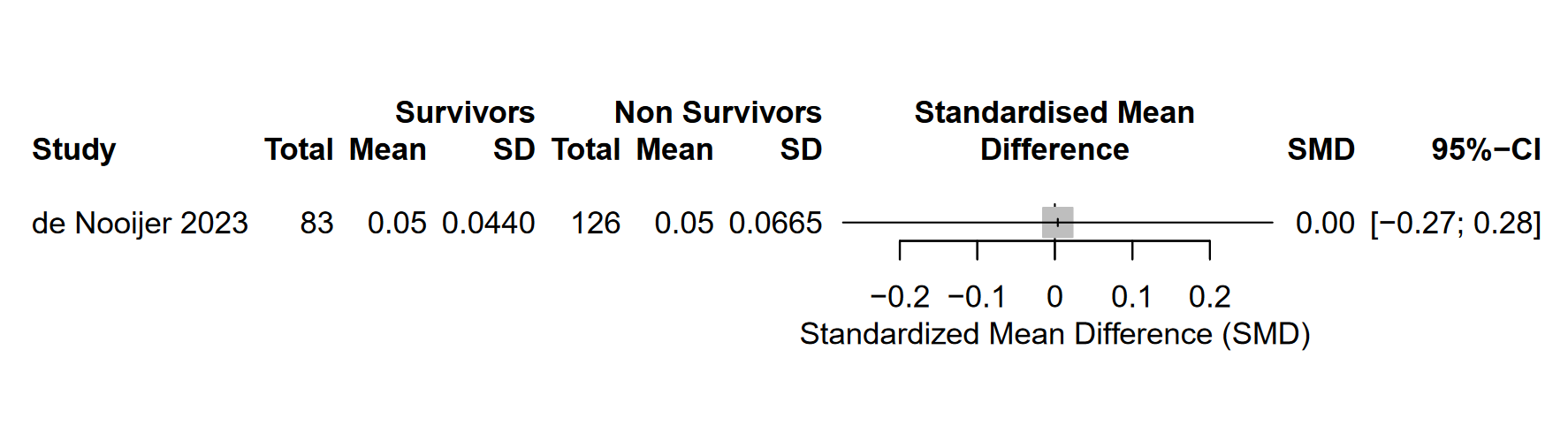C3c  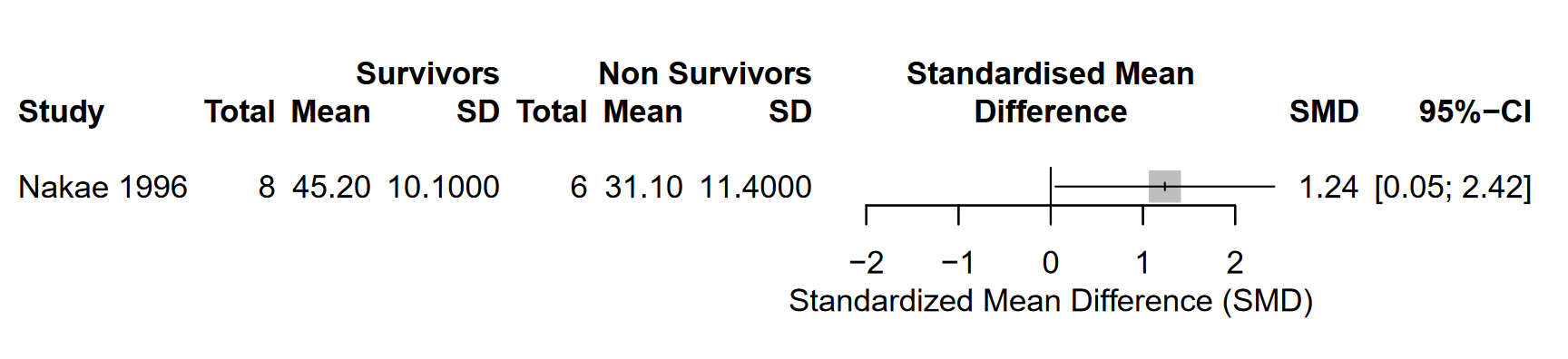  CH50  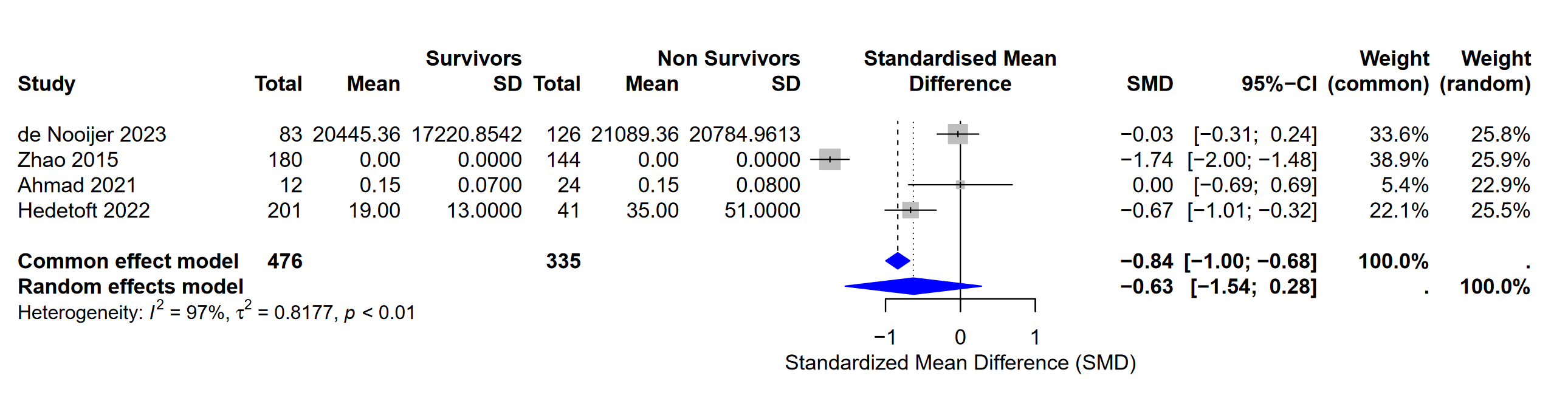MAC  For simplification TCC reported in de Noojer et al and Hedetoft et al articles as well as sC5b-9 reported in Ahmad’s article were considered as same as MAC.  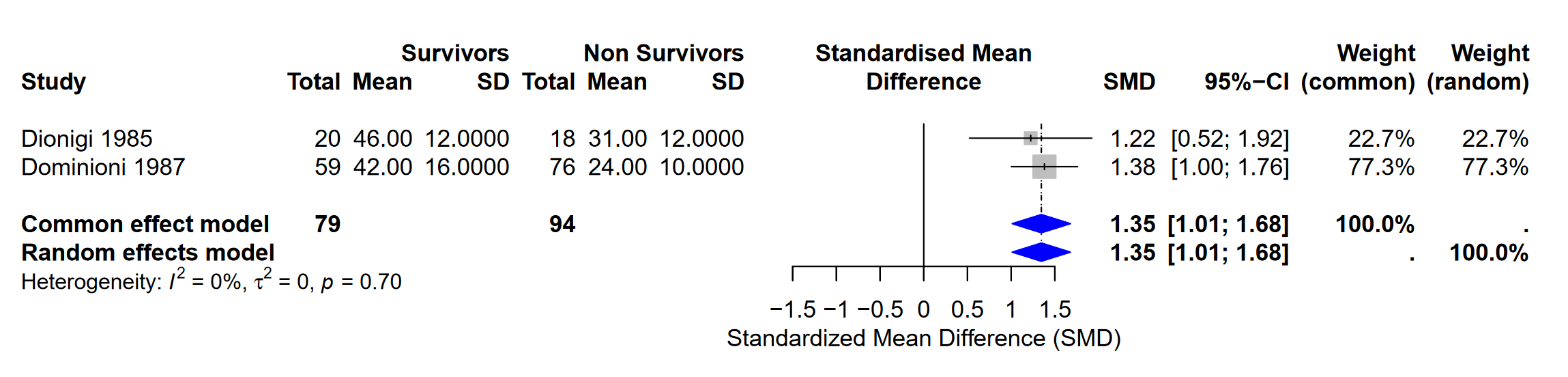  FB  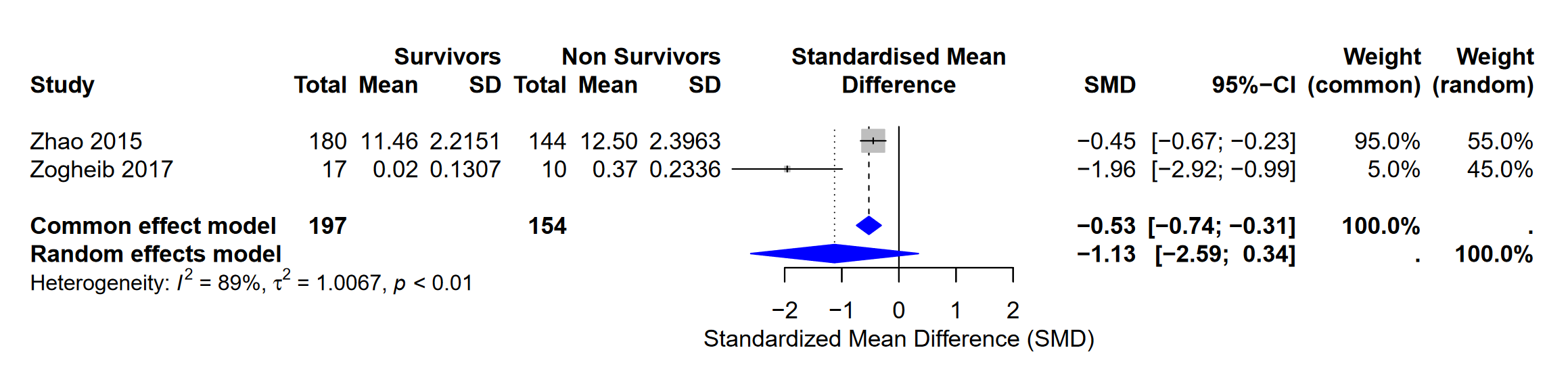  MBL  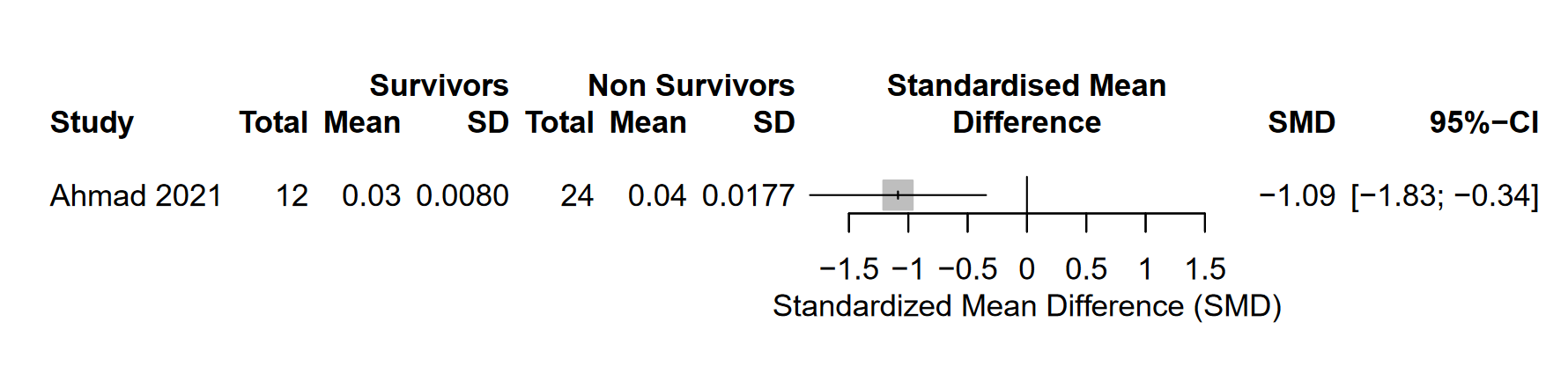  sCD59  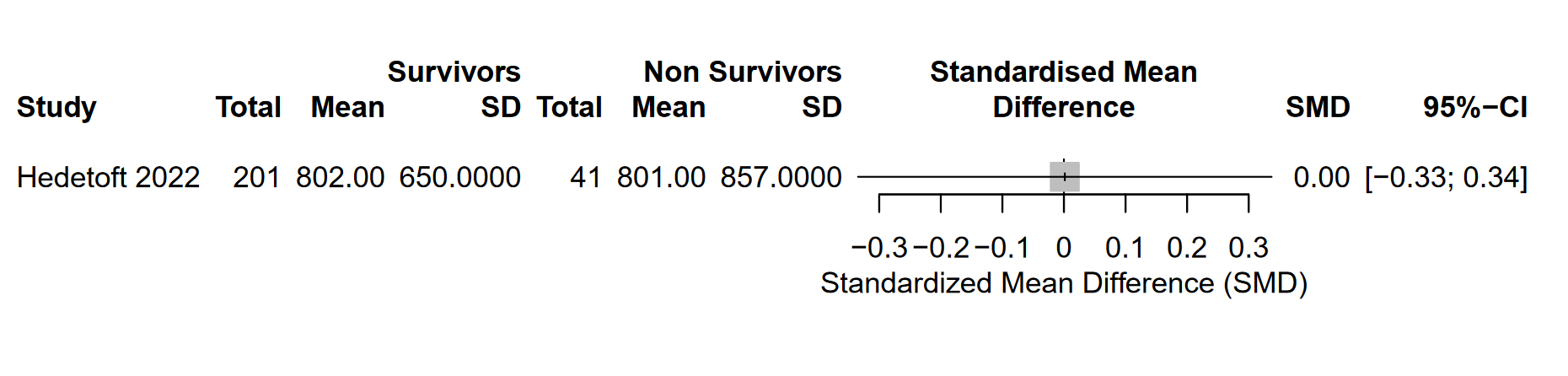  C3dg  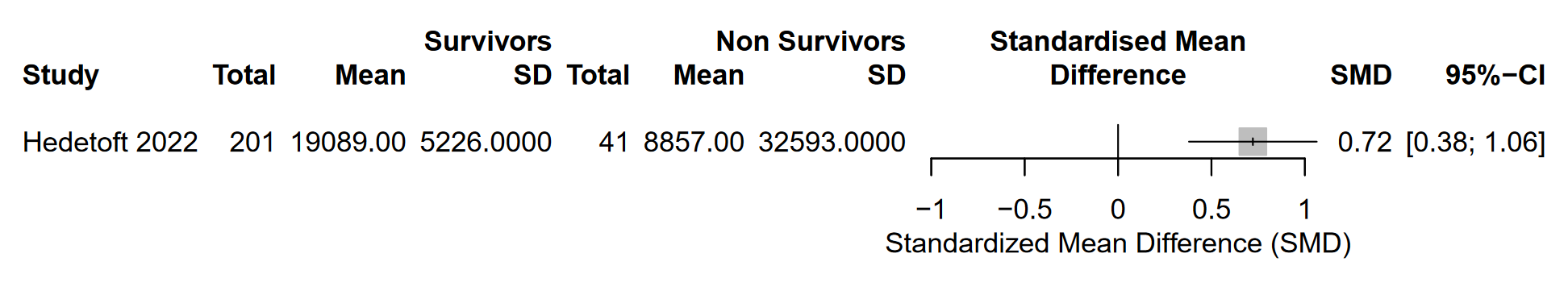  C3bc  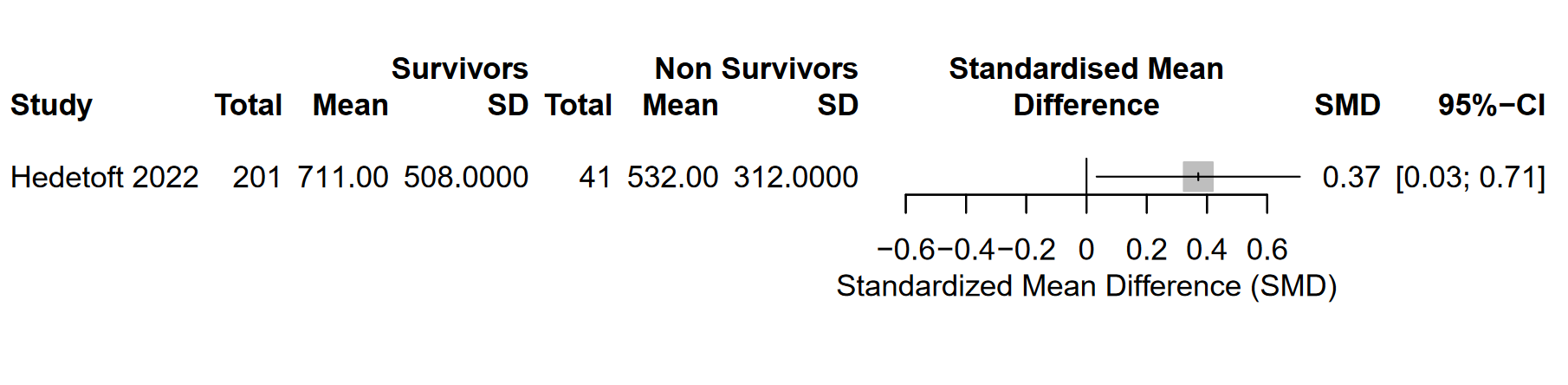  C4c  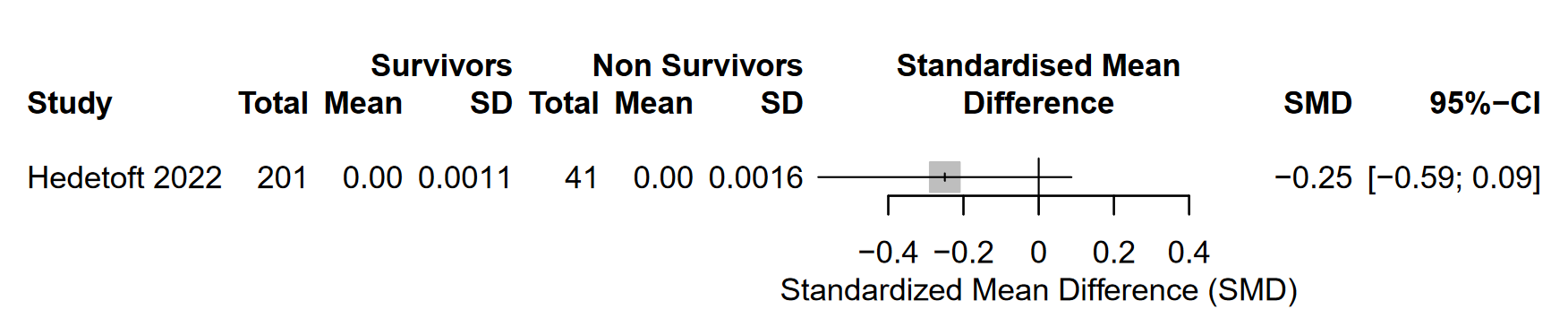  C4d  Supplemental Figure 2: **Meta-analysis of complement proteins in patients with sepsis.** *Forest plots comparing the levels of different complement proteins between survivors and non-survivors in the ICU. Each individual study is represented by a square, with its size proportional to the study's weight in the meta-analysis. Horizontal bars indicate 95% confidence intervals. The blue diamond represents the pooled effect estimate from the random-effects model. Heterogeneity values (I²) are provided for each analysis.* |
| --- |

| 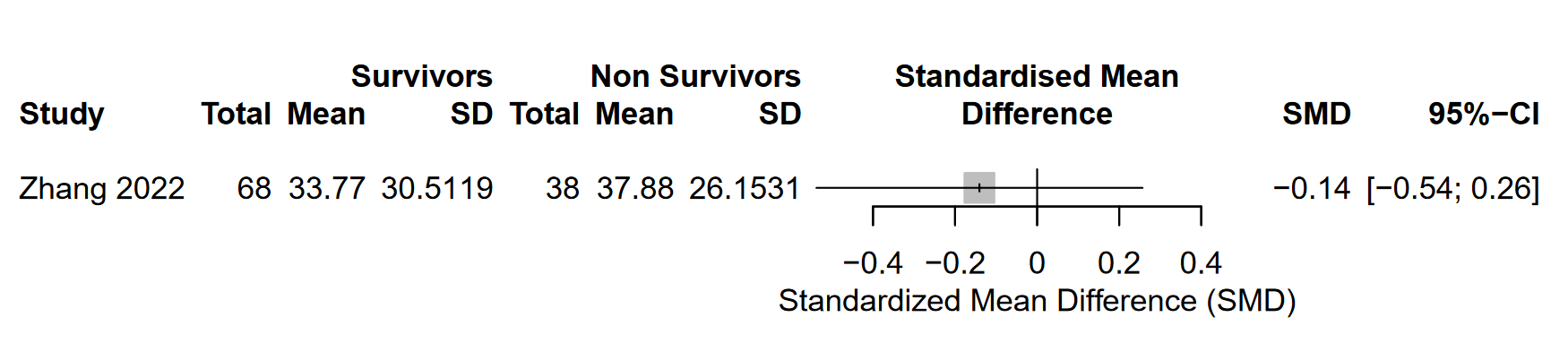  IgA1  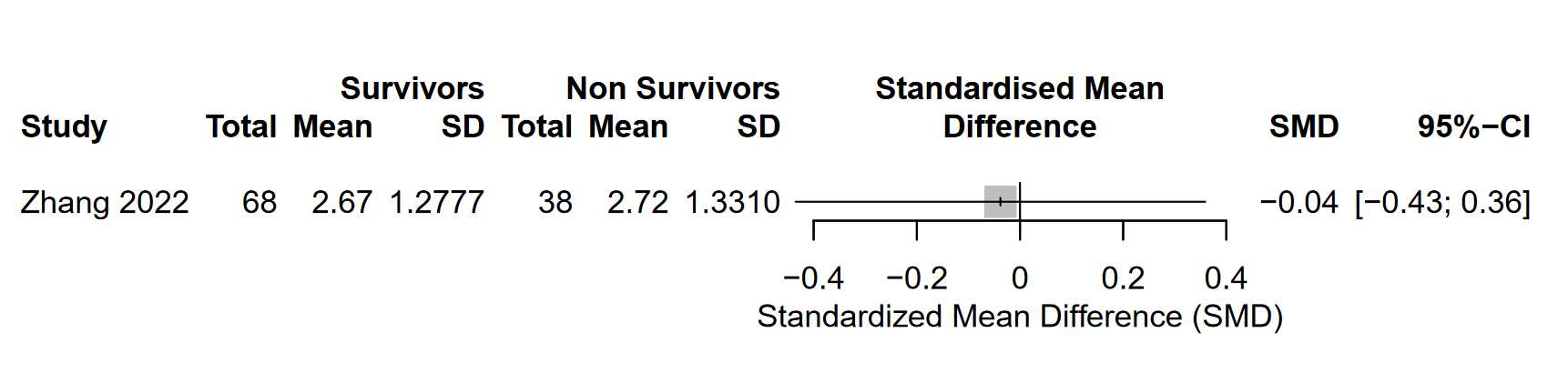  IgA2  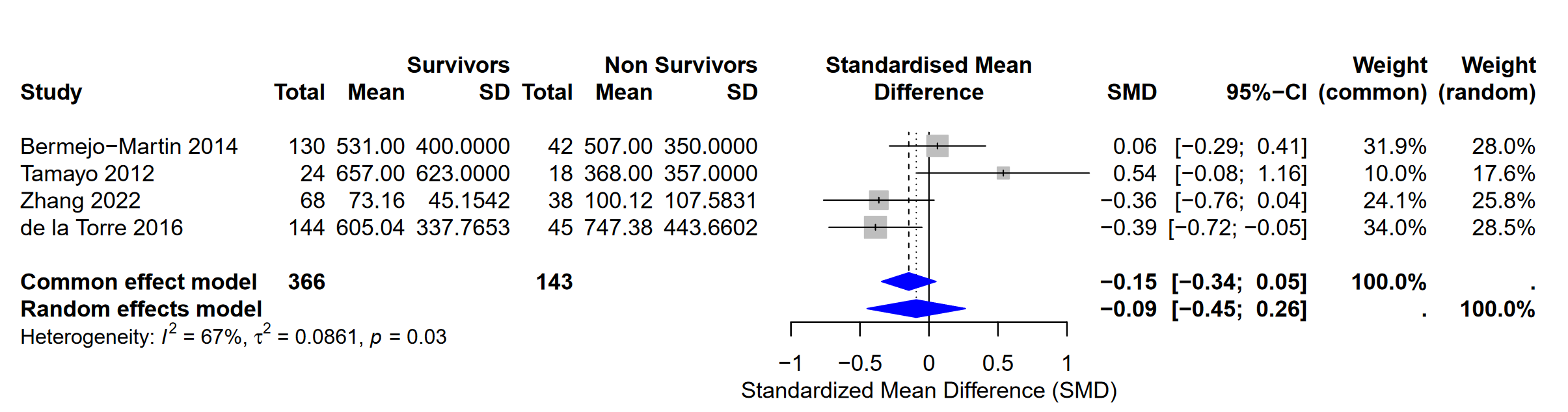  IgG1  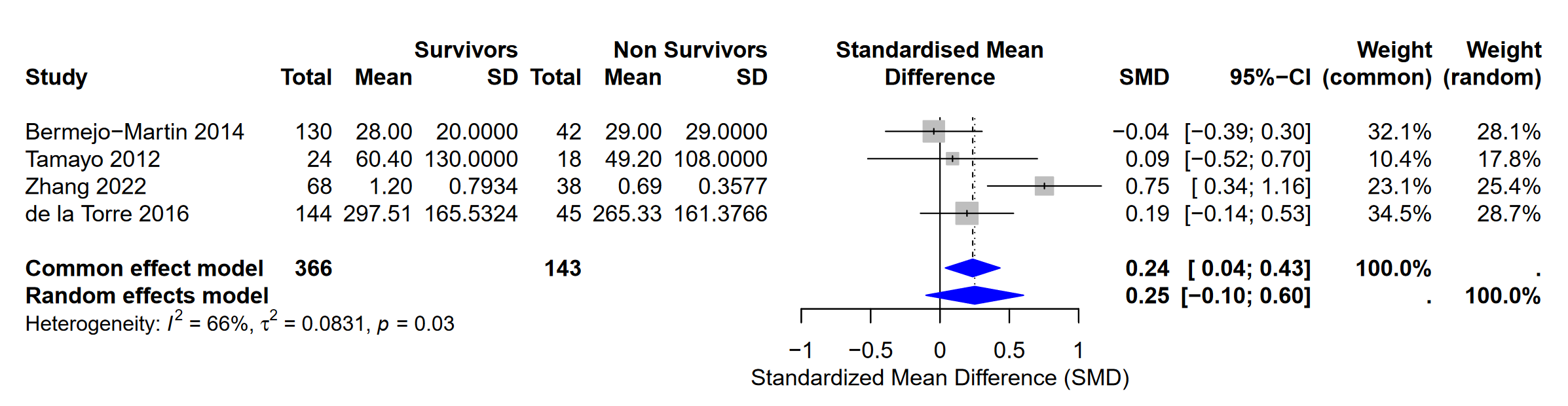  IgG2  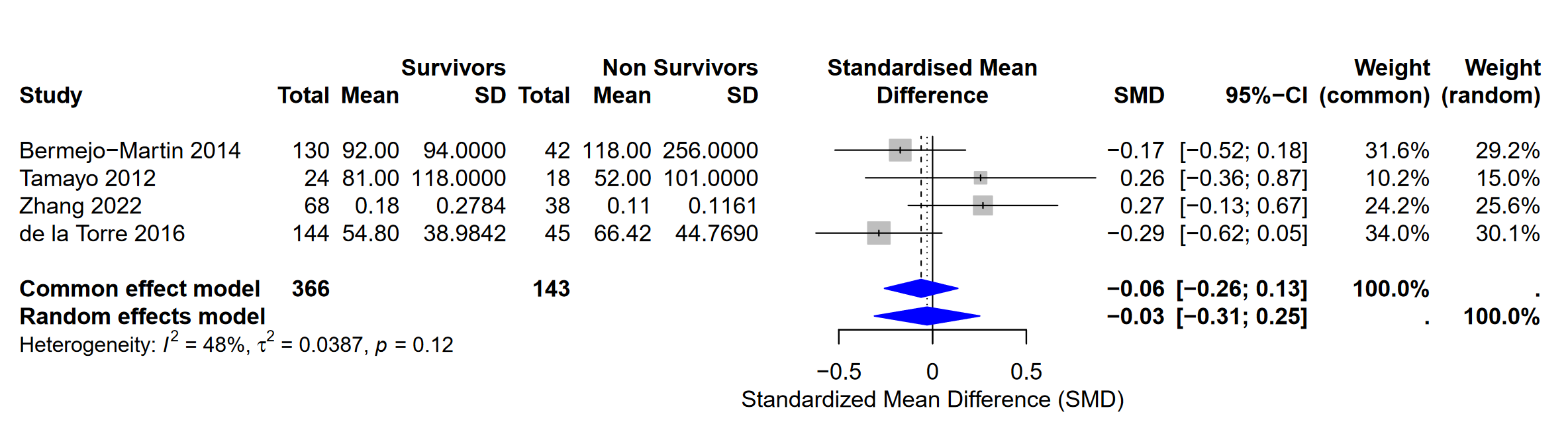  IgG3  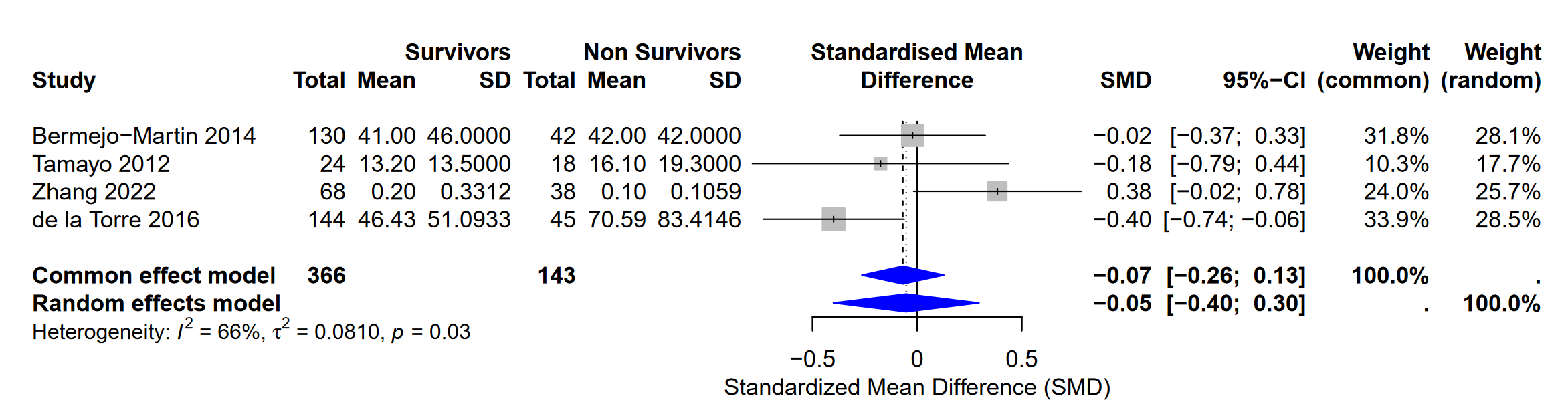  IgG4  Supplemental Figure 3: **Meta-analysis of immunoglobulin in patients with sepsis.** *Forest plots comparing the levels of different complement proteins between survivors and non-survivors in the ICU. Each individual study is represented by a square, with its size proportional to the study's weight in the meta-analysis. Horizontal bars indicate 95% confidence intervals. The blue diamond represents the pooled effect estimate from the random-effects model. Heterogeneity values (I²) are provided for each analysis.* |
| --- |

| 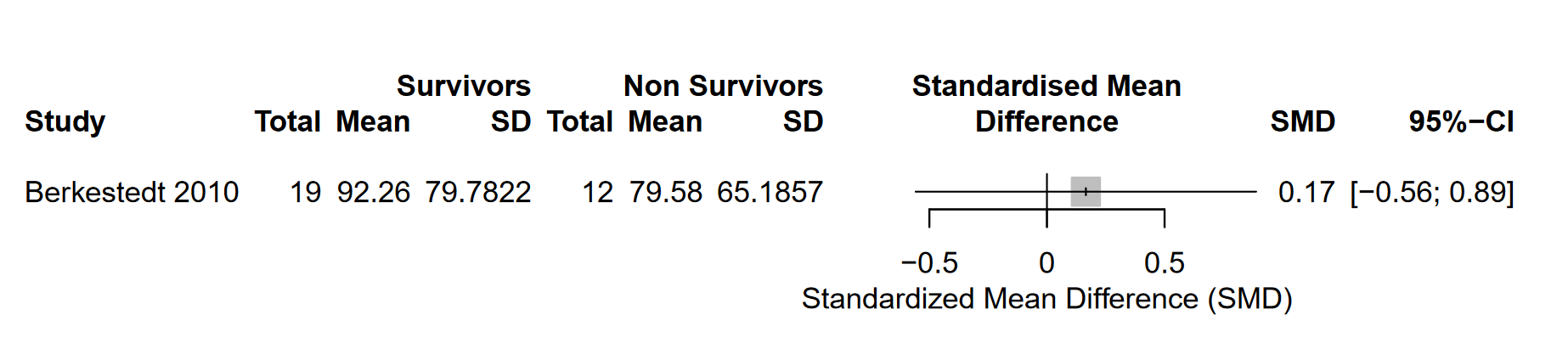  Defensin  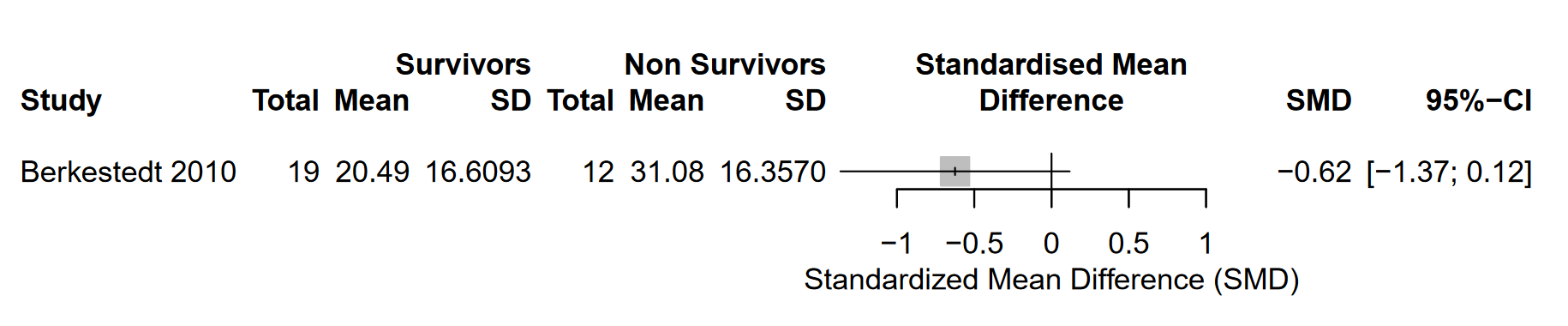  BPI  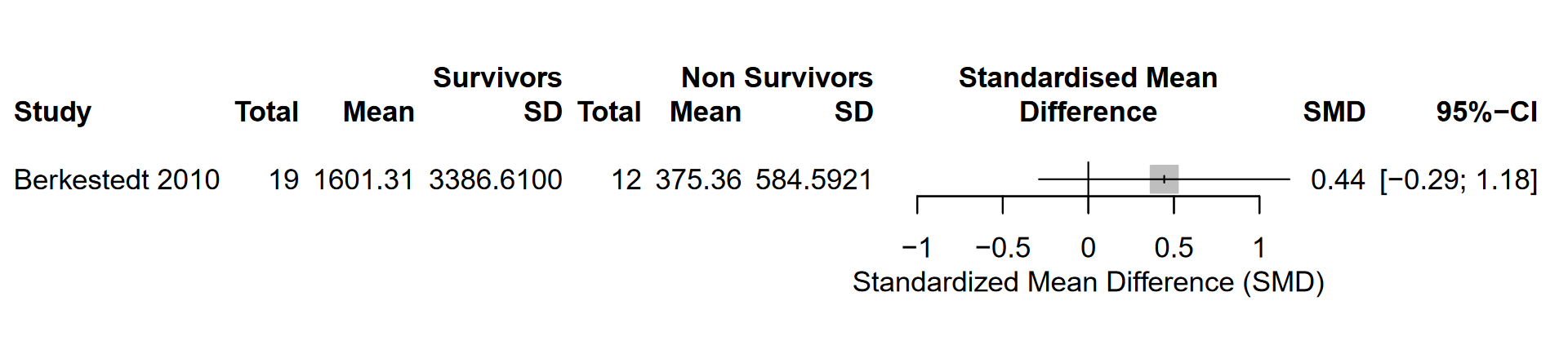  Lactoferrin  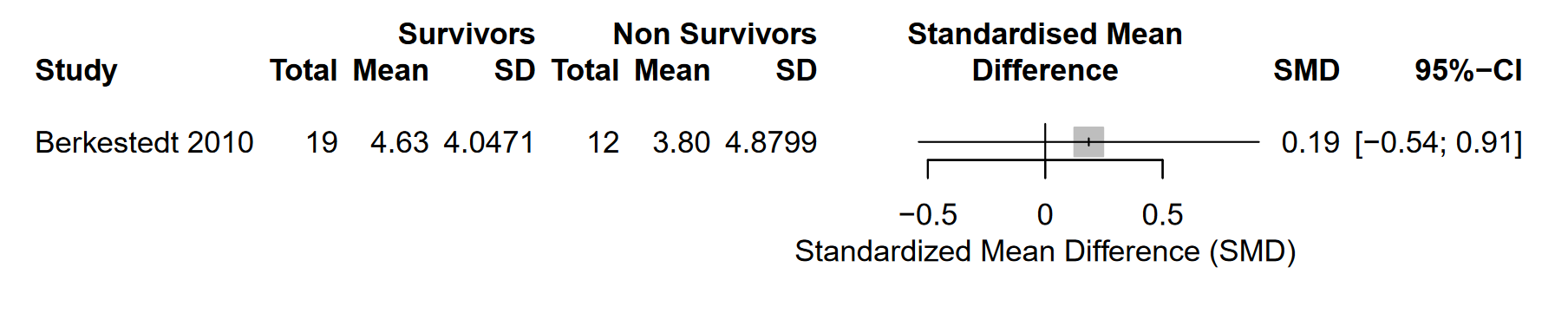  LL-37  Supplemental Figure 4: **Meta-analysis of Anti-Microbial Peptides in patients with sepsis.** *Forest plots comparing the levels of different complement proteins between survivors and non-survivors in the ICU. Each individual study is represented by a square, with its size proportional to the study's weight in the meta-analysis. Horizontal bars indicate 95% confidence intervals. The blue diamond represents the pooled effect estimate from the random-effects model. Heterogeneity values (I²) are provided for each analysis.* |
| --- |

| 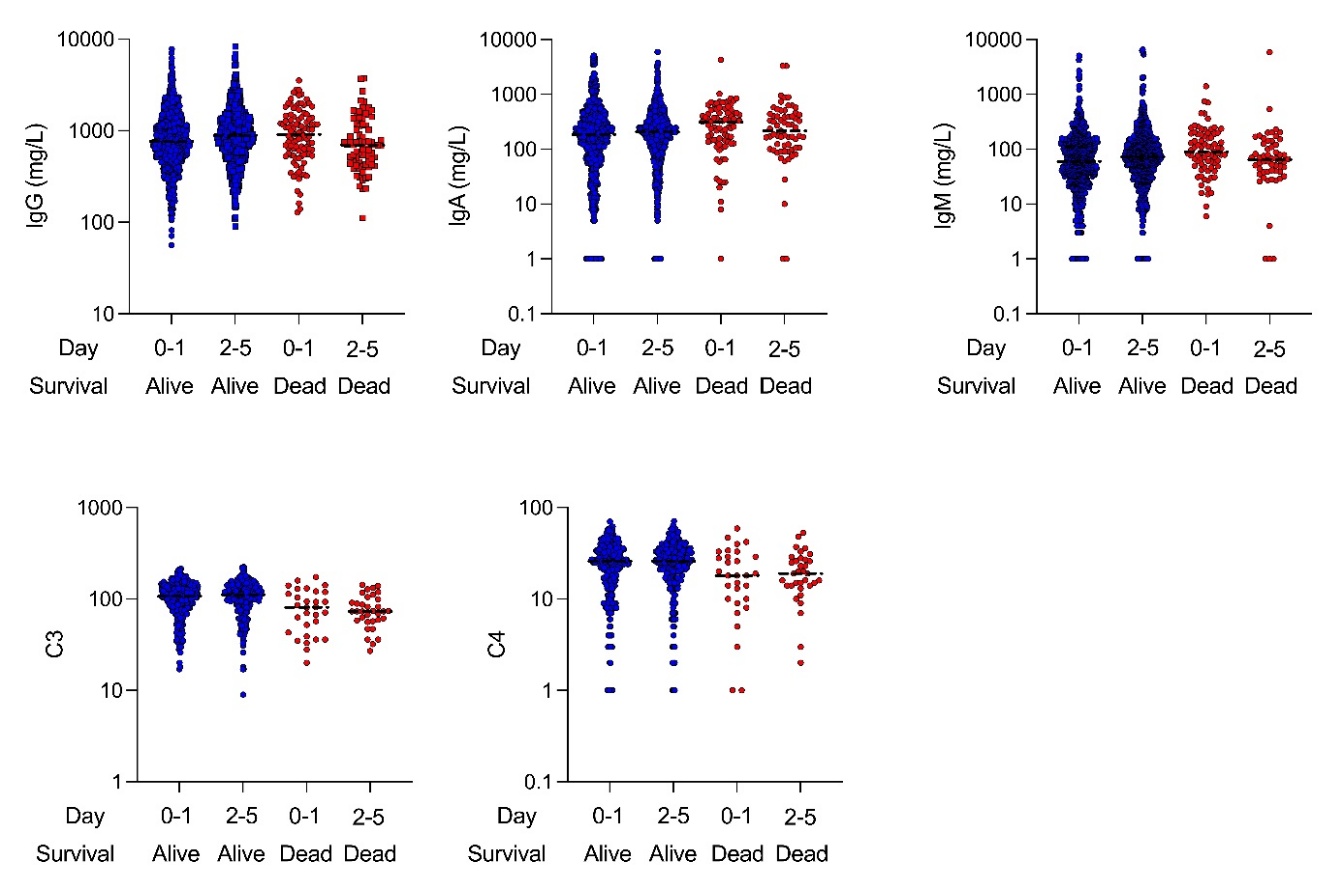  Supplemental Figure 5: **MIMIC’s data**: Boxplots illustrating the levels of immunoglobulins (A-C) and complement proteins (D-E) in critically ill patients stratified by survival status (blue = survivors, red = non-survivors) and time period (0-1 days vs. 2-5 days post-admission). |
| --- |

| **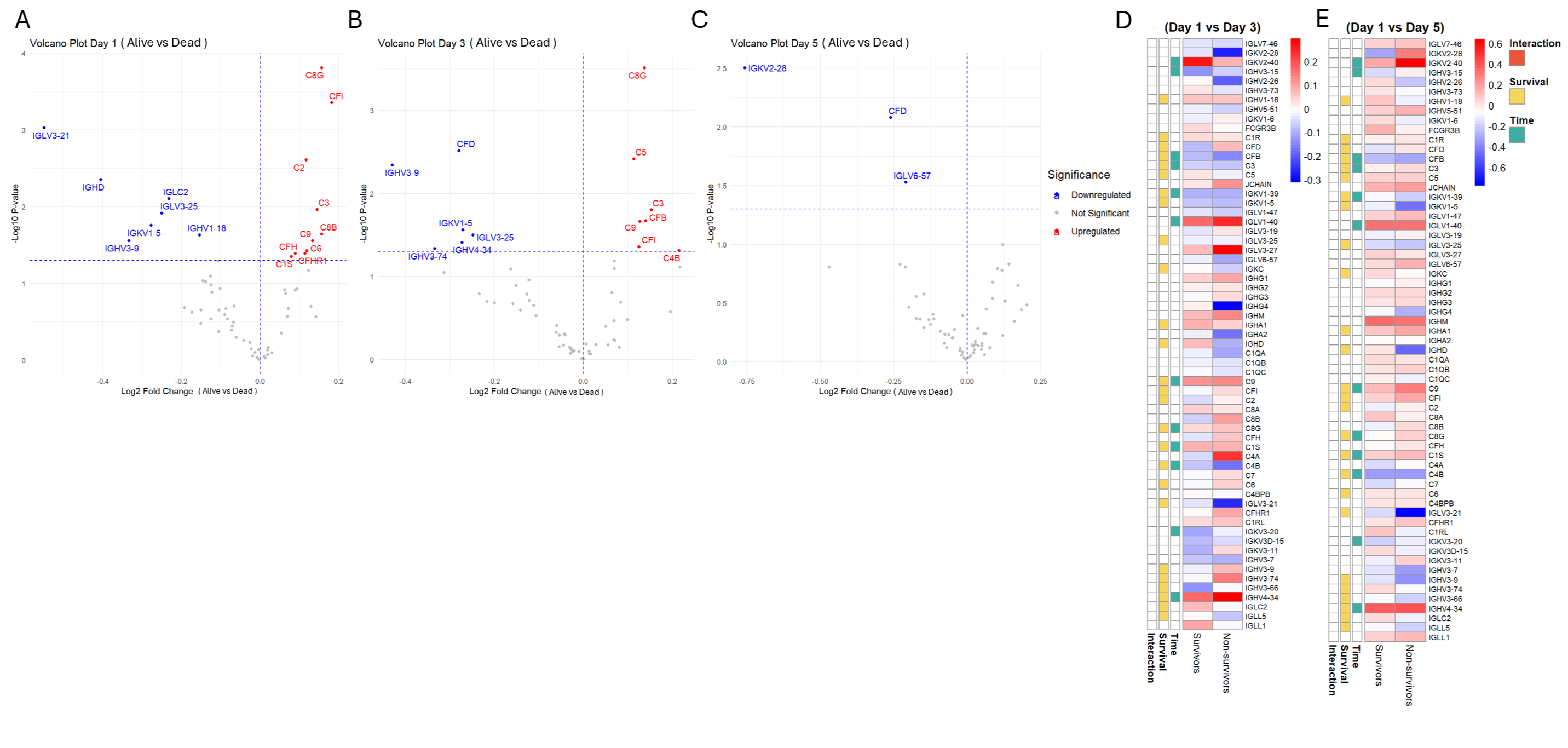**  Supplementary Figure 6: **Proteomic and mass spectrometry data from patients presenting in the ICU with sepsis (adapted from Mi et al. [68])**  **(a–c)** Volcano plots comparing protein abundance between survivors and non-survivors on Day 1 (a), Day 3 (b), and Day 5 (c) after ICU admission. The x-axis represents the log₂ fold change (Alive vs. Dead), where positive values indicate higher abundance in survivors and negative values indicate higher abundance in non-survivors. The y-axis shows the –log₁₀(p-value). Proteins significantly overexpressed in survivors are shown in red, while those overexpressed in non-survivors are shown in blue. Grey points represent proteins with non-significant differences.  **(d–e)** Heatmaps showing temporal log₂ fold changes in protein abundance from Day 1 to Day 3 (d) and Day 1 to Day 5 (e), displayed separately for survivors and non-survivors. Rows represent individual proteins; columns represent timepoint comparisons. The color gradient reflects the direction and magnitude of change (red = increase; blue = decrease). Colored bars on the left indicate significant effects based on two-way ANOVA: green for time effect, yellow for survival effect, and orange for time × survival interaction.  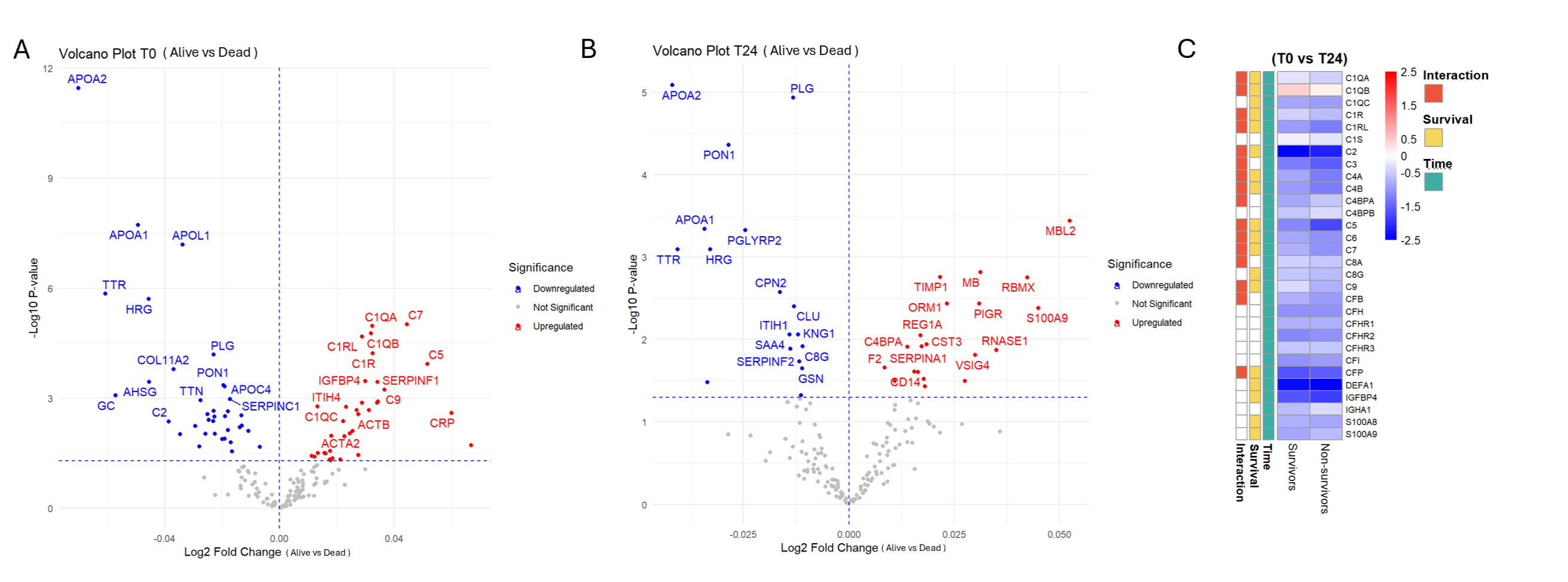  Supplementary Figure 7: **Proteomic Data from Sepsis Patients Presenting to the Emergency Department (adapted from Langley et al. [70])**  **(a)** Volcano plot comparing plasma protein abundance between survivors and non-survivors at baseline (T0), corresponding to the time of Emergency Department admission, prior to antibiotic or fluid administration. The x-axis represents the log₂ fold change (Alive vs Dead), where positive values indicate higher abundance in survivors, and negative values indicate higher abundance in non-survivors. The y-axis displays –log₁₀(p-value). Proteins significantly overexpressed in survivors are shown in red, and those overexpressed in non-survivors in blue. Grey points represent proteins with non-significant differences.  **(b)** Volcano plot at 24 hours post-presentation (T24), showing differential protein abundance between survivors and non-survivors after initial interventions. Axis conventions are the same as in panel (a).  **(c)** Heatmap showing longitudinal changes in plasma protein abundance from T0 to T24 in survivors and non-survivors. Each row corresponds to a protein from the complement system, immunoglobulins, or antimicrobial peptides. The color gradient represents the log₂ fold change (red = increased abundance; blue = decreased abundance). Left-side annotations indicate statistical significance from two-way ANOVA: green = time effect, yellow = survival effect, orange = time × survival interaction. |
| --- |

| 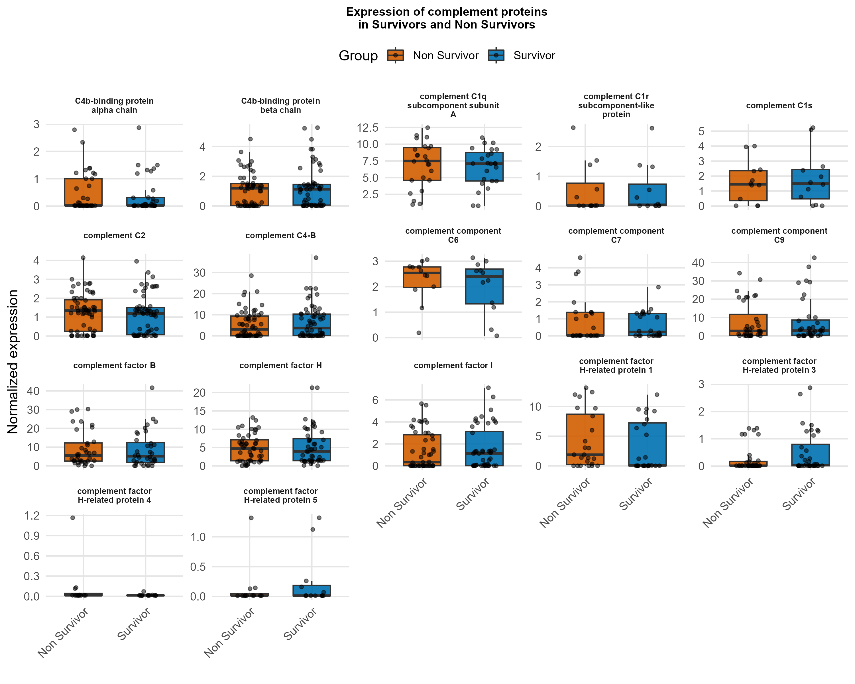  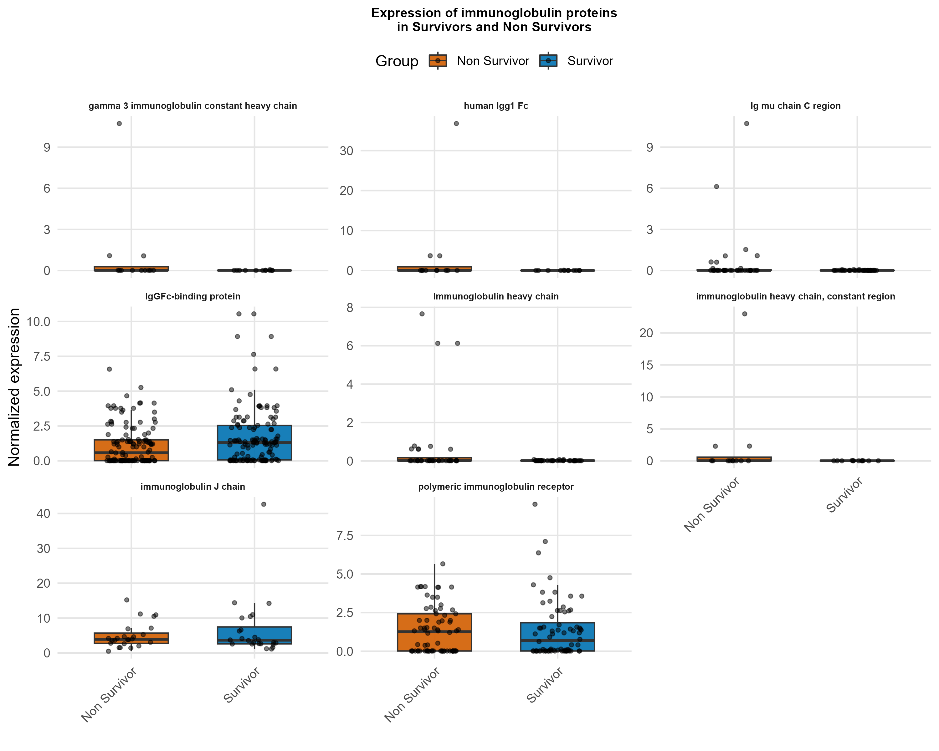  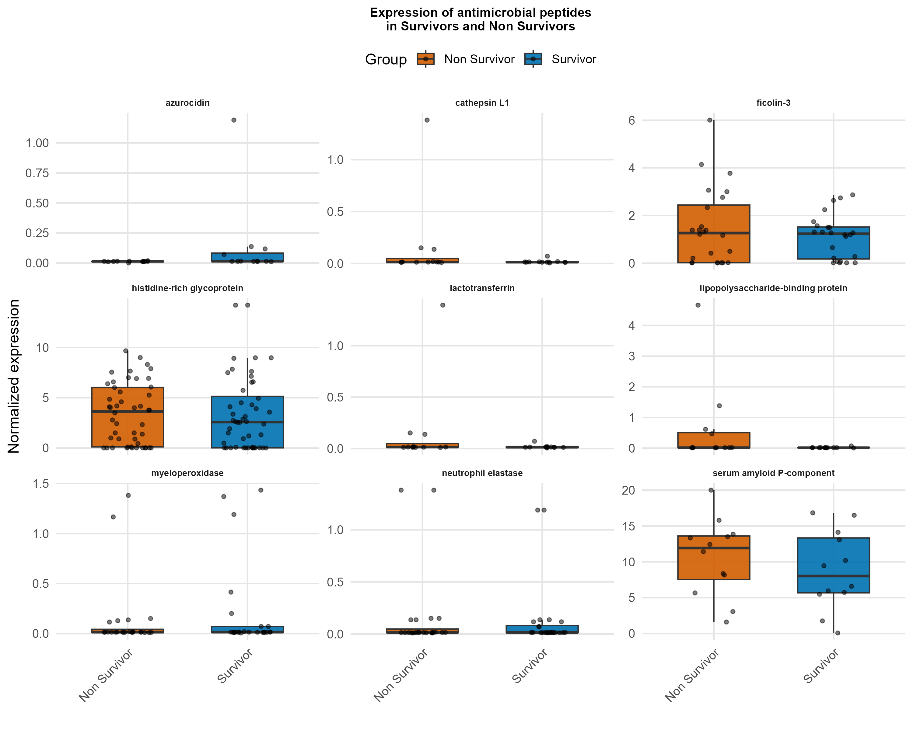  Supplemental Figure 8: **Plasma Proteomic Analysis on ICU Admission (adapted from DeCoux et al. [69])**  **(a)** *Complement protein expression in sepsis survivors and non-survivors.* Box plots display the distribution of normalized expression levels for complement-related proteins in plasma samples collected at ICU admission. While some effector components of the terminal complement cascade (e.g., C9, C7, C2) appear visually elevated in non-survivors, only **complement C5** was significantly different between groups according to an unpaired two-sample t-test (*p* = 0.042).  **(b)** *Immunoglobulin protein expression in survivors and non-survivors.* Box plots showing plasma levels of immunoglobulin-related proteins, including IgG, IgA, and IgM components. Survivors exhibited higher expression of regulatory and transport proteins such as the immunoglobulin J chain, polymeric immunoglobulin receptor, and **IgGFc-binding protein**, the latter of which reached statistical significance (*p* = 0.006). **Immunoglobulin heavy chain** also showed a significant difference (*p* = 0.040).  **(c)** *Antimicrobial peptide and related protein expression.* Box plots presenting expression levels of selected antimicrobial peptides and innate immune mediators, including azurocidin, cathepsin L1, ficolin-3, and myeloperoxidase.  Among the proteins tested, the following reached statistical significance between survivors and non-survivors:   - **Tenascin C** (*p* = 0.017) - **Coagulation factor X** (*p* = 0.020) - **Adenosine deaminase CECR1** (*p* = 0.036) - **Pregnancy zone protein** (*p* = 0.036) - **Neuropilin-1** (*p* = 0.042)   Statistical comparisons were performed using unpaired t-tests on normalized protein expression values. |
| --- |

| C3  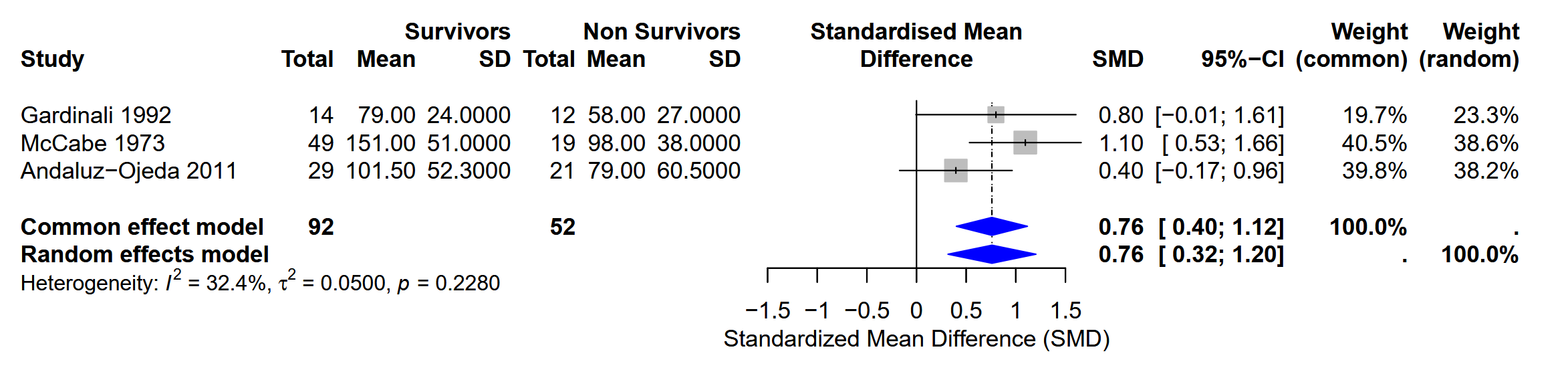  C3a  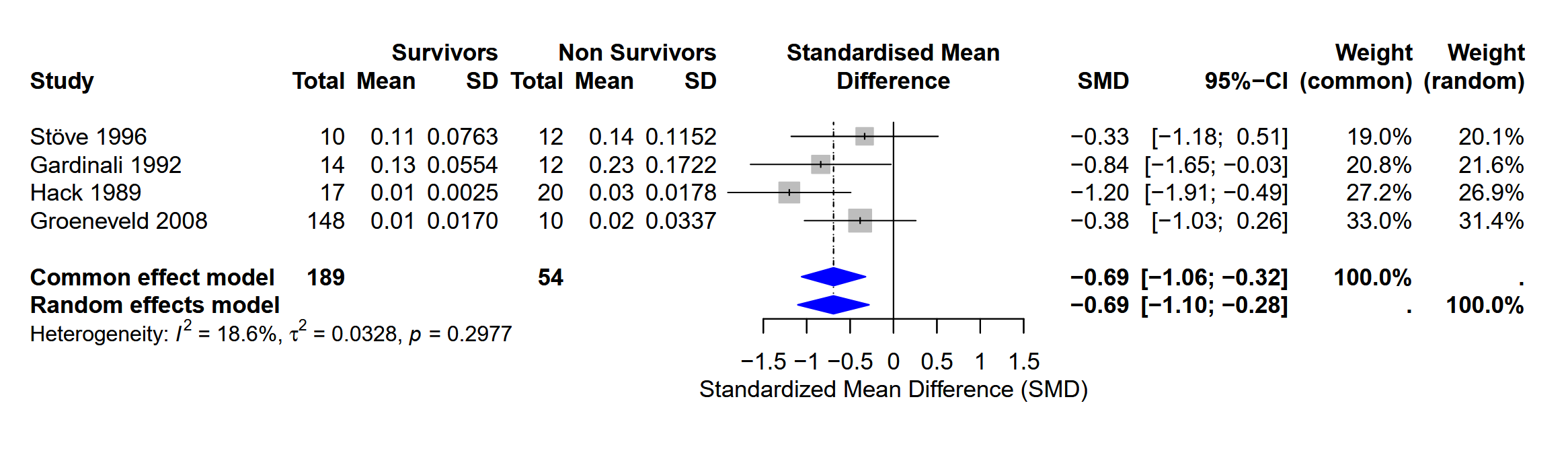  C4  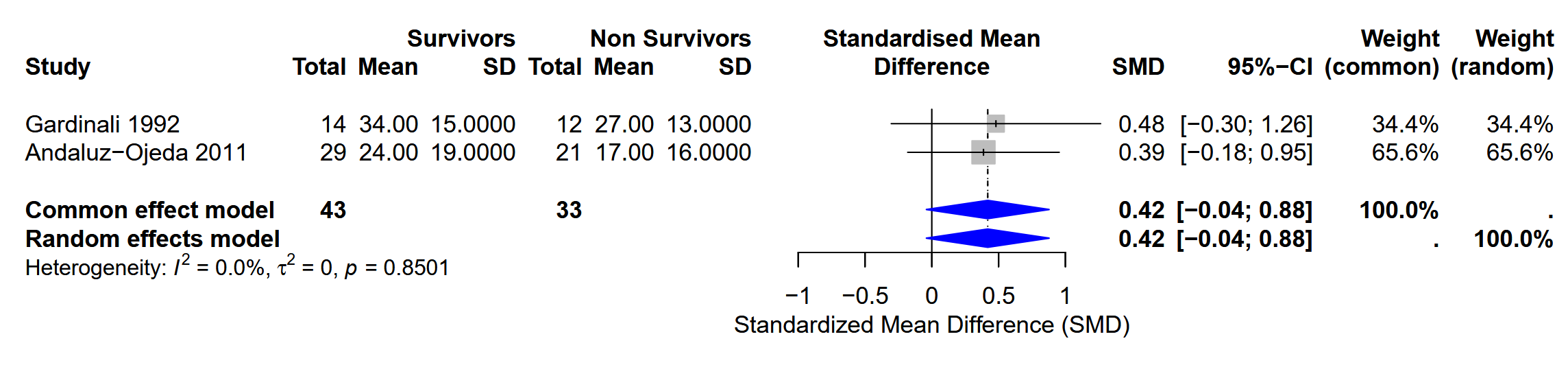  IgA  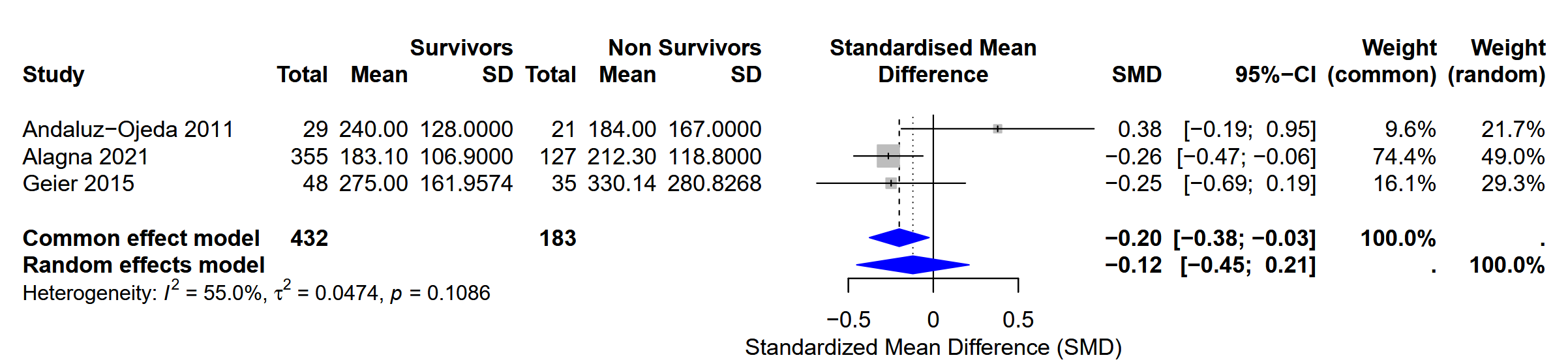  IgG  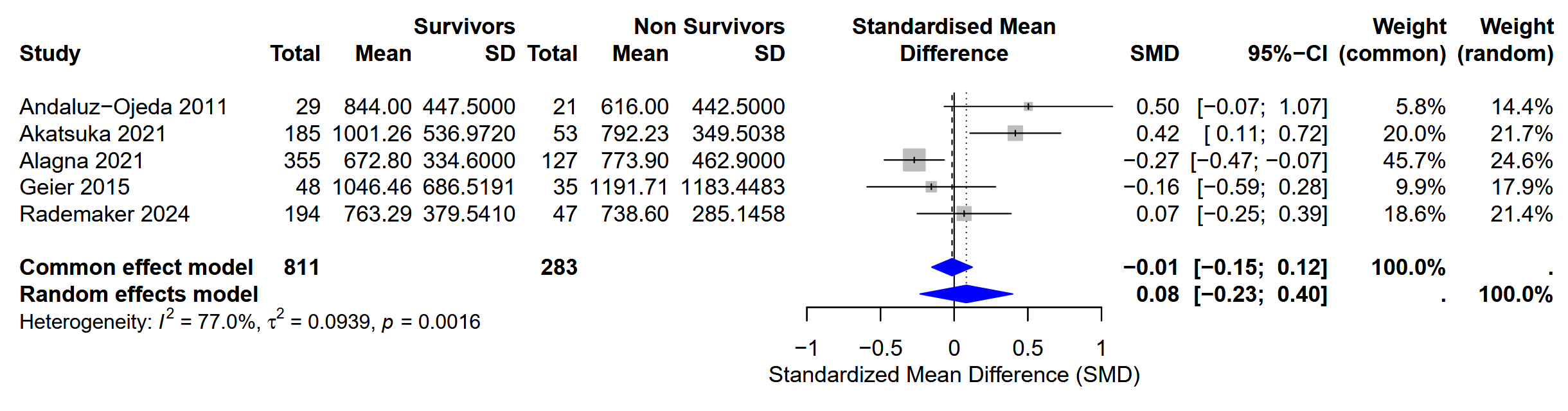  IgM  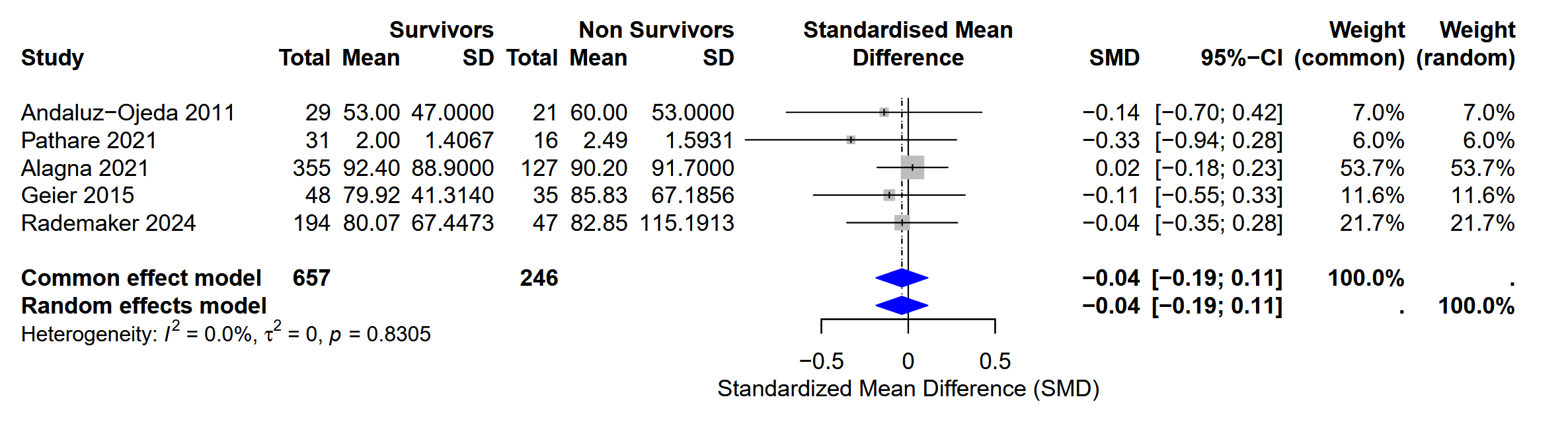  **Supplemental Figure 9**. Sensitivity analysis restricted to studies with protein measurements performed within 24 hours of ICU admission. |
| --- |

| 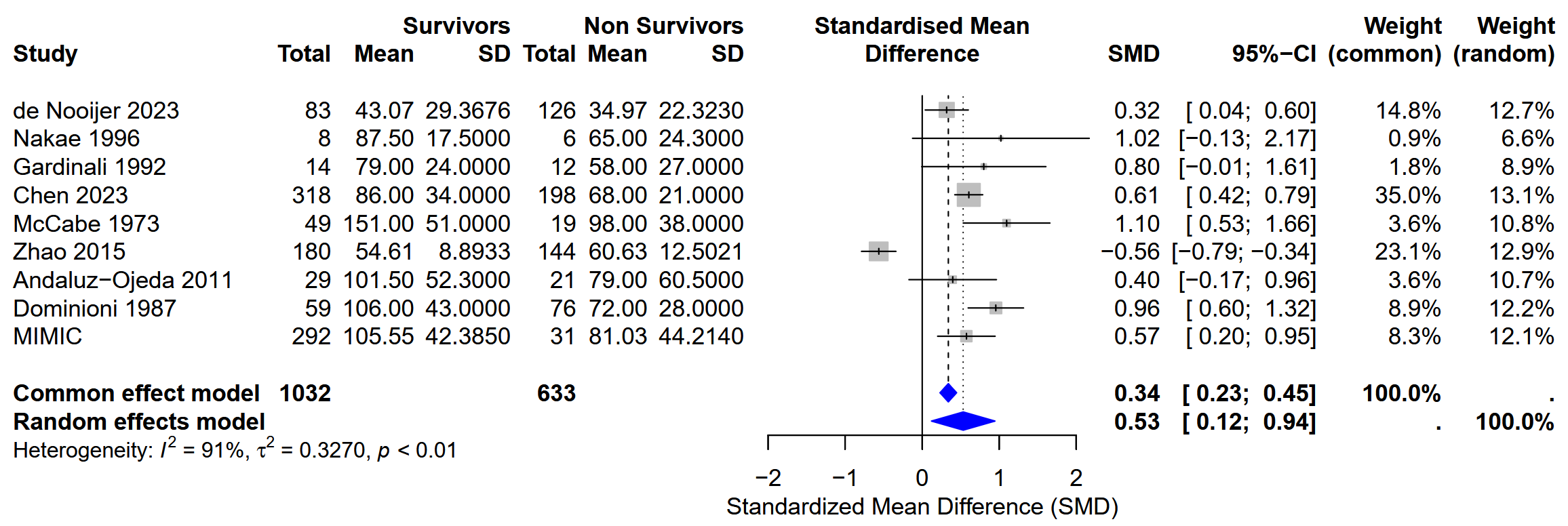  C3  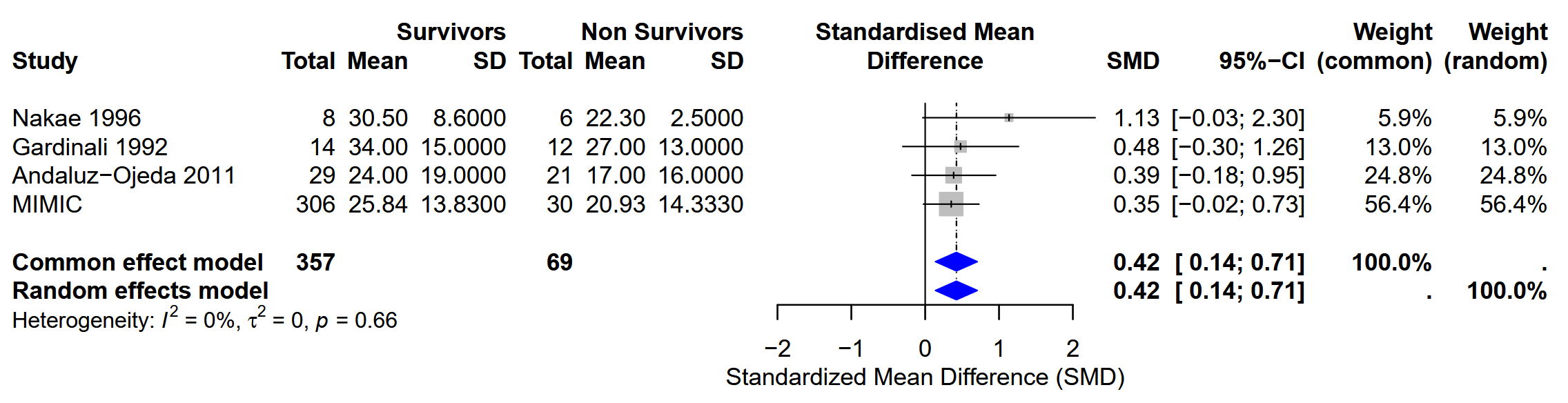  C4  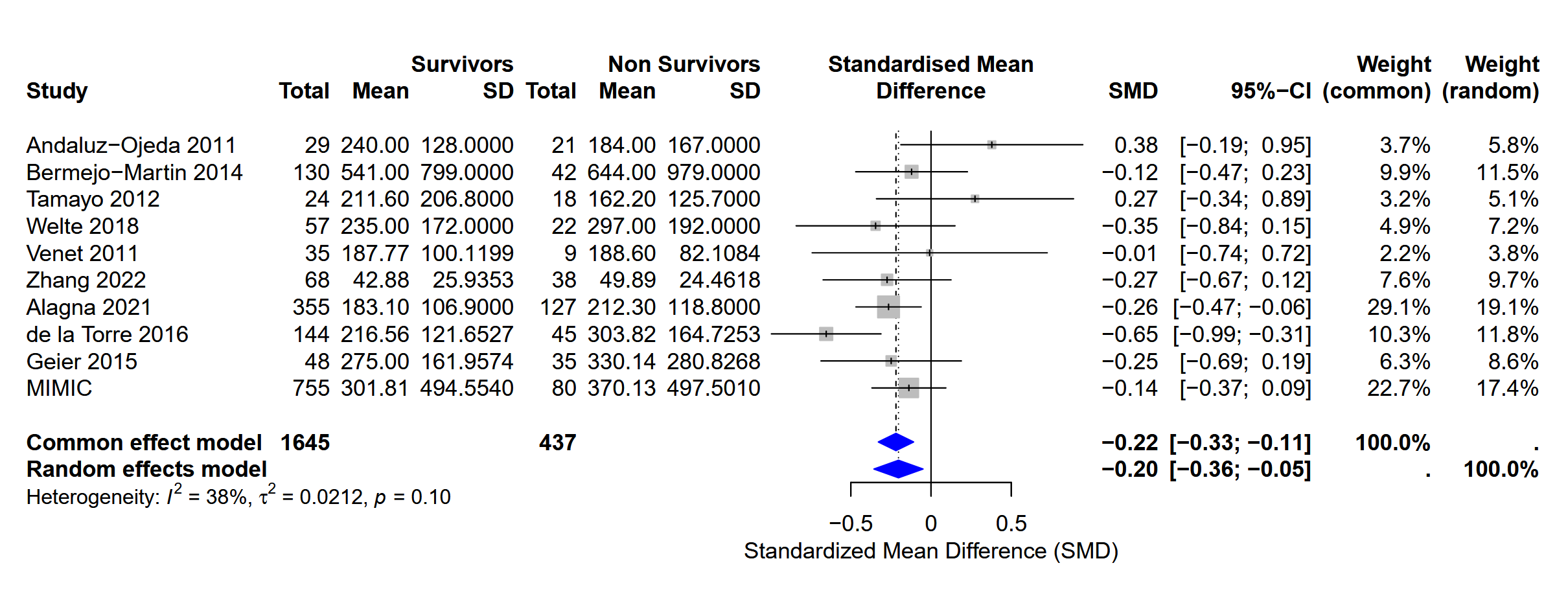  IgA  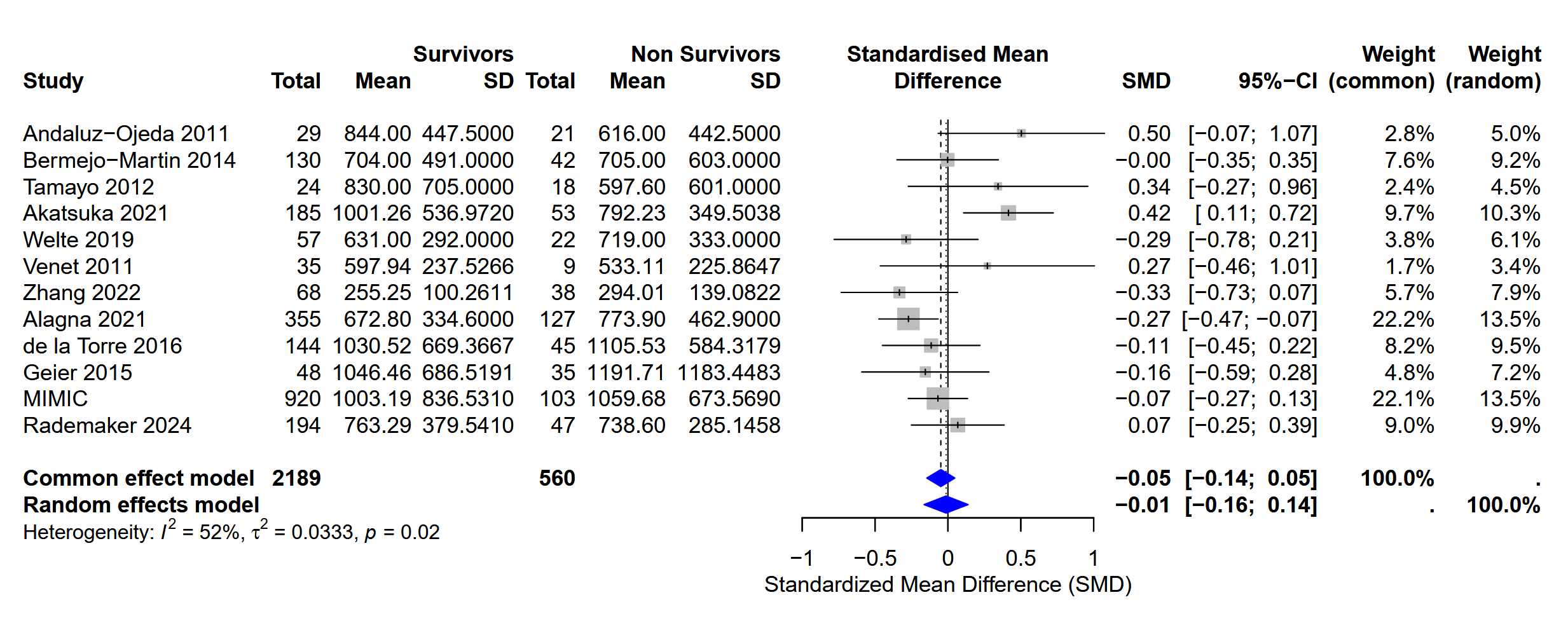  IgG  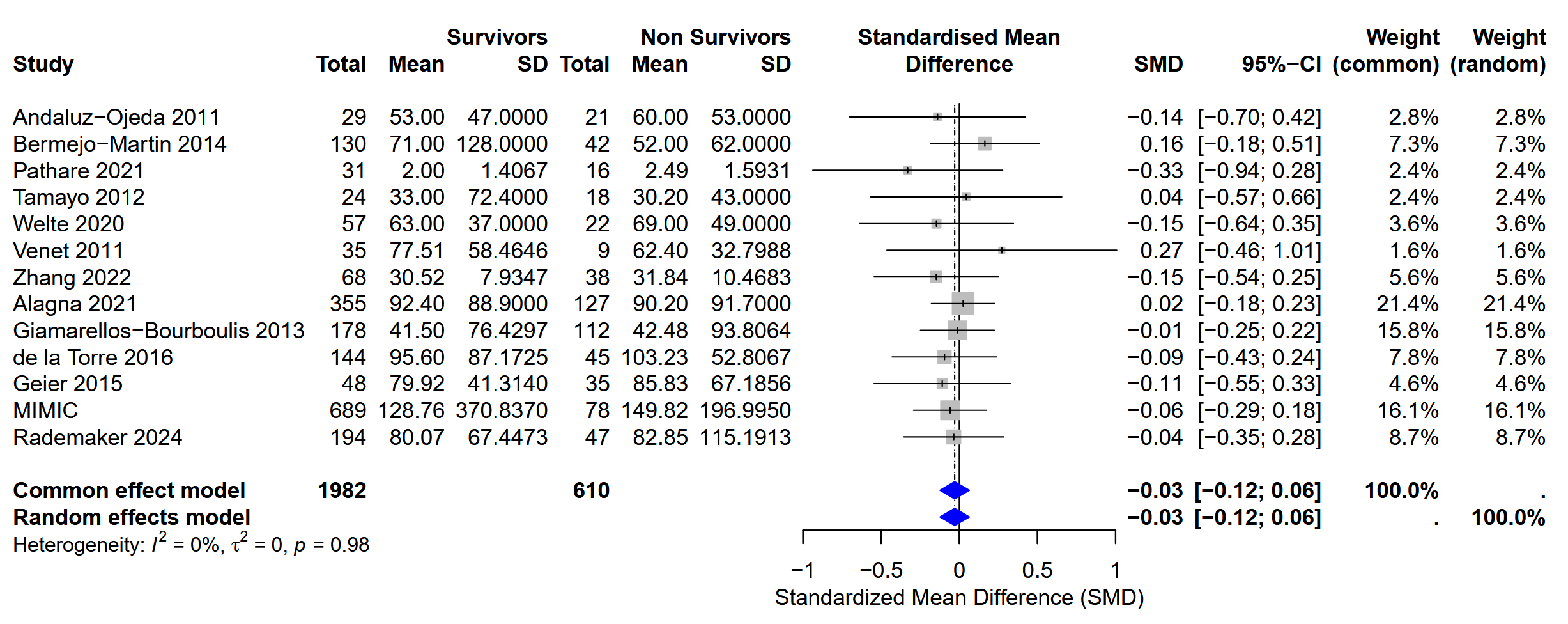  IgM  **Supplemental Figure 10**: Meta analysis of serum complement proteins and immunoglobulins with inclusion of the MIMIC data |
| --- |
